# An improved PKPD modeling approach to characterize the pharmacodynamic interaction over time between ceftazidime/avibactam and colistin from *in vitro* time-kill experiments against multidrug-resistant *Klebsiella pneumoniae* isolates

**DOI:** 10.1128/aac.00301-23

**Published:** 2023-09-08

**Authors:** Romain Aubry, Julien Buyck, Laure Prouvensier, Jean-Winoc Decousser, Patrice Nordmann, Sebastian G. Wicha, Sandrine Marchand, Nicolas Grégoire

**Affiliations:** 1 Université de Poitiers, PHAR2, Inserm U1070, Poitiers, France; 2 Laboratoire de Toxicologie-Pharmacologie, CHU de Poitiers, Poitiers, France; 3 Department of Bacteriology and Infection Control, University Hospital Henri Mondor, Assistance Publique - Hôpitaux de Paris, Créteil, France; 4 Faculté de Médecine de Créteil, Ecole nationale vétérinaire d'Alfort (EnvA), EA 7380 Dynamyc Université Paris - Est Créteil (UPEC), Créteil, France; 5 Medical and Molecular Microbiology Unit, Faculty of Science and Medicine, University of Fribourg, Fribourg, Switzerland; 6 Swiss National Reference Center for Emerging Antibiotic Resistance (NARA), University of Fribourg, Fribourg, Switzerland; 7 Institute for Microbiology, University of Lausanne and University Hospital Centre, Lausanne, Switzerland; 8 Department of Clinical Pharmacy, Institute of Pharmacy, University of Hamburg, Hamburg, Germany; Providence Portland Medical Center, Portland, Oregon, USA

**Keywords:** antimicrobial combination, PKPD modeling, ceftazidime/avibactam, colistin

## Abstract

In contrast to the checkerboard method, bactericidal experiments [time-kill curves (TKCs)] allow an assessment of pharmacodynamic (PD) interactions over time. However, TKCs in combination pose interpretation problems. The objective of this study was to characterize the PD interaction over time between ceftazidime/avibactam (CZA) and colistin (CST) using TKC against four multidrug-resistant *Klebsiella pneumoniae* susceptible to both antibiotics and expressing a widespread carbapenemase determinant KPC-3. *In vitro* TKCs were performed and analyzed using pharmacokinetic/pharmacodynamic (PKPD) modeling. The general pharmacodynamic interaction model was used to characterize PD interactions between drugs. The 95% confidence intervals (95%CIs) of the expected additivity and of the observed interaction were built using parametric bootstraps and compared to evaluate the *in vitro* PD interaction over time. Further simulations were conducted to investigate the effect of the combination at varying concentrations typically observed in patients. Regrowth was observed in TKCs at high concentrations of drugs alone [from 4 to 32× minimum inhibitory concentrations (MIC)], while the combination systematically prevented the regrowth at concentrations close to the MIC. Significant synergy or antagonism were observed under specific conditions but overall 95%CIs overlapped widely over time indicating an additive interaction between antibiotics. Moreover, simulations of typical PK profile at standard dosages indicated that the interaction should be additive in clinical conditions. The nature of the PD interaction varied with time and concentration in TKC. Against the four *K. pneumoniae* isolates, the bactericidal effect of CZA + CST combination was predicted to be additive and to prevent the emergence of resistance at clinical concentrations.

## INTRODUCTION

Among the most difficult to treat bacterial infections, those caused by carbapenem-resistant enterobacterales (CRE), which include carbapenemase-producing *Klebsiella pneumoniae* (KPC), constitute a major threat (1). Ceftazidime/avibactam (CZA) is a recent beta-lactam/beta-lactamase inhibitor displaying activity against many types of multidrug-resistant (MDR) Gram-negative bacteria including KPC producers (2). Ceftazidime is hydrolyzed in the presence of KPC enzymes, but its activity can be restored by avibactam (AVI) due to the inhibition of beta-lactamases (3). Despite its limited use, *in vitro* and *in vivo* resistances to CZA have been described in *K. pneumoniae* (4 – 6). To prevent and/or cure emerging antimicrobial resistance, antibiotic combination therapy is one of the strategies (7). In this context, old antibiotics such as polymyxins [colistin (CST) and polymyxin B] have regained interest to increase the antimicrobial panoply and possibly prevent emergence of resistance. Previous *in vitro* studies have reported heterogeneous synergy rates of polymyxins in combination with many antibiotics against *K. pneumoniae* (8 – 10) but only few studies have considered the combination with CZA (11, 12). Because of the diversity of combinations and bacterial strains, it is necessary to develop suitable methodology for selecting the best drug combinations.

Different definitions of synergy can be found, some are based on differences in combination effects compared to monotherapy (e.g., 2 log10 CFU decrease at a given time) (11) and others are based on mathematical/statistical criteria of variation in pharmacodynamic parameters (e.g., Greco interaction parameters significantly different from 0) (13). Traditionally, pharmacodynamic (PD) interactions of combined antibiotics are evaluated by *in vitro* checkerboard experiments, visual evaluation of the bacterial growth at 24 h, calculation of the fractional inhibitory concentration index (FICI) and classified as synergy, additivity or antagonism (14). However, several drawbacks have been reported for this methodology since the effect, measured at a single time point, is based on a discrete output (growth/no growth of the bacteria) and does not inform about the emergence of resistances over time (15, 16).

*In vitro* time-kill curves (TKCs) have been extensively used to study the time-course effect of antibiotics and bacterial regrowth (17 – 20). The longitudinal data from TKCs in combination can be analyzed using semi-mechanistic pharmacokinetic/pharmacodynamic (PKPD) models (21 – 24). As is the case with checkerboard data, it may be useful to classify the PD interactions observed in these experiments as synergistic, additive, or antagonistic. However, compared to the checkerboards, there is an additional degree of complexity since the interactions are also evaluated over time. The use of PD models considering TKC data longitudinally (with differential equations) makes it possible to take into account the correlations existing between successive CFU counts from the same tube. This provides a gain in statistical power over analysis approaches that would consider the times independently of each other.

Mathematical characterization of the type of interaction requires to predict the expected effect under an assumption of additivity of the effects of each antibiotic [e.g., Loewe additivity (25) or Bliss independence (26)]. Among the few papers that reported TKCs with antibiotics in combination and characterized the type of interaction using PKPD models, the criterion for model selection was based on a statistical comparison of the interaction parameter (27 – 30) and was derived from the Greco synergy model (13, 31). However, a significant change of the interaction parameter does not necessarily translate into a significant change of the combination effect. To our knowledge, no study has characterized a PD interaction based on the statistical comparison of effects over time. Such an analysis would require comparing the confidence intervals of the effect predicted under the additivity assumption to those obtained experimentally in combination, and where an overlap of these intervals would mean a non-significant difference (i.e., an additive interaction) (32). The construction of these confidence intervals is based on the uncertainty of the parameter estimates, which can be calculated by different methods including the asymptotic variance-covariance matrix (33), the parametric or non-parametric bootstraps (34), and the sampling importance Resampling (SIR) (35).

The objective of this study was to characterize the PD interaction over time between CZA and CST using TKC data against four MDR *K. pneumoniae* isolates expressing the same carbapenemase. A comparison of 95% confidence intervals (95%CIs) between the observed effect in combination and the expected effect under the additivity assumption was performed and further simulations were conducted to investigate the effect of the combination at varying concentrations typically observed in patients.

## MATERIALS AND METHODS

### Antibiotics

Ceftazidime and avibactam were purchased from MedChemExpress (NJ, USA). Colistin sulfate was purchased from Sigma Aldrich (Saint-Quentin-Fallavier, France). Stock solutions of antibiotics (10,240 mg/L) were prepared in sterile water, stored at −80°C, and diluted in Mueller Hinton Broth II cation adjusted (MHB) on the day of experiment to achieve the desired concentrations.

### Strains

Four clinical *K. pneumoniae* isolates, susceptible to CZA and CST, obtained from the National Reference Center for Emerging Antibiotic Resistance (NARA, Fribourg, Switzerland), were selected from its human strain collection from 2019 to 2021. They were used for *in vitro* time-kill experiments for an in-depth evaluation of the CZA + CST combination.

### *In vitro* susceptibility testing

Minimum inhibitory concentrations (MIC) of CST and of CZA in the presence of 4 mg/L of avibactam were determined for each strain by microdilution, according to the EUCAST guidelines (36).

### Static time-kill experiments

*In vitro* time-kill experiments included a growth control, single drug experiments with CST ranging from 0.125 to 32 times the MIC with twofold serial dilution or CZA (with fixed avibactam concentration of 4 mg/L) ranging from 0.125 to 8 times the MIC. Combinations of both drugs were tested at several concentrations (at least 16 combinations of CZA + CST, ranging from 0.25 + 0.25 to 2 + 2 times the MIC, were systematically performed for each strain).

One microliter of frozen bacteria was taken from −80°C storage and incubated overnight in 10 mL of MHB at 35°C with constant shaking (130 rpm). A 200 µL volume of this bacterial suspension was diluted in 10 mL of MHB and pre-incubated (2 h, 35°C, 130 rpm) to reach exponential growth phase. The optical density (600 nm) was adjusted between 0.08 and 0.11, corresponding to approximately 10^8^ CFU/mL bacterial density. Then, 50 µL of the bacterial suspension was added to each culture tube to achieve a 5 × 10^5^ CFU/mL inoculum. The preparation (10 mL total volume) containing the antibiotics at targeted concentrations was incubated at 35°C with constant shaking (130 rpm) for 30 h. Samples were taken at 0, 2, 4, 8, 24, and 30 h, serially diluted in NaCl 0.9%, and plated on MHA using an easySpiral automatic plater (Interscience, France). Plates were incubated 24 h at 35°C and CFU were enumerated using a SCAN 300 colony counter (Interscience, France). The lower limit of quantification was 200 CFU/mL. Each experimental condition was tested one to four times.

### Sequencing before/after antibiotic exposure

All isolates were sequenced before drug exposure in order to investigate their resistance genes. Briefly, the genomes were sequenced using Illumina technology, as previously described (37). Then, the genomes were assembled with Shovill v1.0.4 (38) and the genetic background and resistome were identified using Kleborate version 2.0.0 (39). In case of regrowth at 24 h, a volume of 1 mL from culture tubes with CZA alone, CST alone, or the combination was sampled and centrifuged at 2,500 RCF for 6 min. Supernatant was removed and bacterial pellets were picked up and cultured to investigate gene mutations following the procedure described above. The growth control was also sampled at 24 h to exclude random mutations unlinked to antibiotic treatment.

### PKPD modeling of single drug effects and expected additivity in TKC

**Fig 1:**
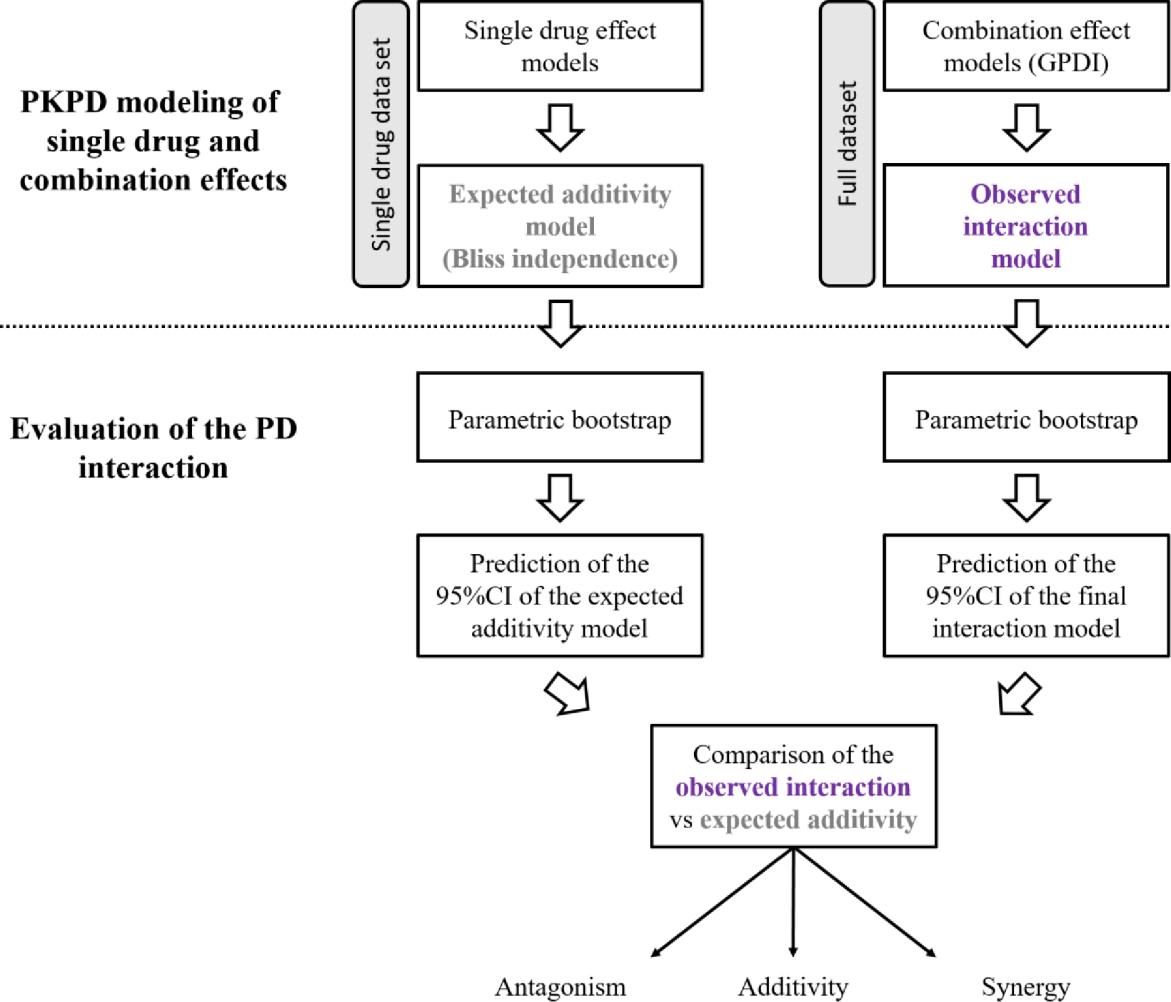
Flowchart for the development of the PKPD model and the evaluation of the PD interaction.

The procedure for the development of the PKPD model and the evaluation of the PD interaction is illustrated in Fig. 1. Data sets were prepared using R software (version 3.6.2) and the parameters of the model were estimated using NONMEM software (version 7.4) with the Laplacian algorithm. Data below the limit of quantification were handled using Beal’s M3 method (40). The only random effect estimated in the model was the residual error in CFU prediction, considered additive in log_10_ scale. Evaluation and selection of the model were based on objective function value (OFV), goodness of fit plots [including individual fits, predicted vs observed CFU, and visual predictive checks (VPCs)], and accuracy of parameter estimates (relative standard errors).

In the first step of the analysis, only the time-kill data from the growth control and from the single drug experiments were used to determine the single drug effect models. The inoculum size was estimated and a logistic growth model with a single homogeneous bacterial population was used to describe the growth control experiments. Ceftazidime (in the presence of 4 mg/L avibactam) and CST were assumed to induce bacterial killing according to a sigmoidal Emax model (equation 1).


(1) $$k_{drug}=\frac{{Emax}_{drug}\times C_{drug}^{\gamma_{drug}}}{{EC50}_{drug}^{\gamma_{drug}}+C_{drug}^{\gamma_{drug}}}$$

where *k* (*h* ^−1^) is the killing rate constant of the corresponding drug, Emax (*h* ^−1^) is the maximum kill rate of the drug, *C* (mg/L) is the drug concentration, EC_50_ (mg/L) is the drug concentration required to achieve 50% of Emax, and *γ* is the sigmoidicity parameter for the single drug effect.

In order to explain the bacterial regrowth observed in TKCs, it was assumed that bacteria could have developed adaptive resistances under antibiotic exposure using an empirical adaptation model. This process was modeled using two virtual compartments describing the fraction of bacteria in the adapted or in the non-adapted state to the drug (18) (equations 2 and 3). At the start of the experiment, all bacteria were assumed nonadapted. No reversal of adaptive resistance was considered since the drug concentrations were assumed constant during the experiment.


(2) $$\frac{{dAR}_{off\_drug}}{dt}=-k_{on\_drug}\times {AR}_{off\_drug}$$

(3) $$\frac{{dAR}_{on\_drug}}{dt}=k_{on\_drug}\times {AR}_{off\_drug}$$

where AR_off_ and AR_on_ are, respectively, the fractions of bacteria nonadapted and adapted to the drug and *k*_on_ (*h* ^−1^) is the rate constant for the development of adaptive resistance.

After testing different adaptation models on Emax and EC_50_, the effect of the bacterial adaptation was represented using an empirical model (equation 4). The Emax parameter of the drug was proportionally reduced to the fraction of adapted bacteria until reaching a residual effect when all bacteria reached the adapted state.


(4) $$k_{drug}=\frac{{Emax}_{drug}\times (1-{ARmax}_{drug}\times {AR}_{on\_drug})\times C_{drug}^{\gamma_{drug}}}{{EC50}_{drug}^{\gamma_{drug}}+C_{drug}^{\gamma_{drug}}}$$

where ARmax (%) is the maximal reduction of the maximum drug effect when all the bacteria were in the adapted state and other parameters are previously described.

Finally, CFU were calculated at each timepoint according to equation 5:


(5) $$\frac{dB}{dt}=\left(k_{g}\times \left(1-\frac{B}{10^{Bmax}}\right)-k_{drug}\right)\times B$$

where *B* (log_10_ CFU/mL) is the bacterial count determined at time *t*, *k*_g_ (*h* ^−1^) is the bacterial growth rate constant, Bmax (log_10_ CFU/mL) is the maximal capacity supported by the medium, and *k*_drug_ is defined in equation 4.

The same structure of the model was used to describe the effect of CZA or CST against the four isolates but different parameter values were estimated for each one. Finally, the expected additivity model, describing the effect of the combination under the additivity assumption (i.e., in the absence of PD drug interaction), was defined using the Bliss independence criterion (26). The NONMEM control stream of the expected additivity model for the NARA1295 isolate is provided in Supplementary Code S1.

### Construction of the 95%CI of the expected additivity

To assess the uncertainty of the model predictions and build the 95%CI of the expected additivity, a parametric bootstrap was applied with the Stochastic Simulation and Estimation (SSE) script of PsN 2.9.2 (41). A 1,000 data sets were simulated by random draws in the residual variability (there was no other variability in our model) of the expected additivity model, without considering parameter uncertainty. Subsequently, for each simulated data set, the parameters of the expected additivity model were estimated. Runs with unsuccessful minimization or with non-zero gradient at last iteration were excluded. Finally, individual predictions over time were obtained for each simulation and summary statistics (median, 2.5 and 97.5 percentiles) of bacterial counts were calculated over time in order to build the 95%CI.

### PKPD modeling of the observed combination effects in TKC

In the second step of the analysis, combination effect models were developed using all the time-kill data (including growth control, single drug, and combination experiments). The previous structural model for antibiotics alone was kept and the general pharmacodynamic interaction (GPDI) model (equation 6) (42), implemented under the Bliss Independence hypothesis, was added to characterize the PD interactions between drugs.


(6) $$\theta_{INT}=\theta_{mono}\times \left(1+\frac{INT\times C_{perpetrator}^{\gamma INT}}{{EC}_{50\_INT}^{\gamma INT}+C_{perpetrator}^{\gamma INT}}\right)$$

where *θ*_INT_ is the PD parameter of the victim drug (i.e., the drug affected by the interaction) modified in the presence of the perpetrator drug (i.e., the drug responsible for the interaction), *θ*_mono_ is the PD parameter of the victim drug used alone, INT (%) is the maximal fractional change of the affected PD parameter of the victim drug, EC_50_INT_ (mg/L) is the perpetrator drug concentration required to achieve 50% of INT, and *γ*_INT_ is the sigmoidicity parameter for the combination effect.

The perpetrator drug could affect either the Emax, the EC_50_, or the *K*_on_ parameter of the victim drug. When several single interactions significantly decreased the OFV compared to the expected additivity, these interactions were combined in a single model. The selection of the model that best described the observed interaction, so-called observed interaction model, was based on the difference in OFV between models (likelihood ratio test with a first-order risk, alpha, of 5%). The NONMEM control stream of the observed interaction model for the NARA1295 isolate is provided in Supplementary Code S2. Visual predicted checks (VPCs) were drawn based on 1,000 simulations using the observed interaction model, with the residual error as the only random effect. Median, 10th, and 90th percentiles were calculated at each sampling time and VPCs were plotted to assess the ability of the model to describe the observed data.

### Evaluation of the pharmacodynamic interaction in TKC

The 95%CI of the observed interaction was built following the same parametric bootstrap methodology than previously described for the 95%CI of the expected additivity. Both 95%CIs were laid out in the same figure for comparison. If the 95%CI of the observed interaction was lower than the 95%CI of the expected additivity (i.e., the upper boundary of the 95%CI of the observed interaction was under the lower boundary of the 95%CI of the expected additivity), a higher bacterial killing was observed and the combination was considered synergistic. If both 95%CIs overlapped, the interaction was considered additive as no significant difference could be highlighted. If the 95%CI of the observed interaction was higher than the expected additivity (i.e., the lower boundary of the 95%CI of the observed interaction was above the upper limit of the 95%CI of the expected additivity), a lower bacterial killing was observed, and the interaction was considered antagonistic. As the comparison was performed over the total duration of the time-kill, the type of interaction could vary over time.

### Simulation of the combination effect at varying clinical concentrations

In order to evaluate the PD interaction predicted by our model in the case of variable concentrations (and not fixed as in TKCs), the expected effect in combination was simulated at concentrations mimicking the concentrations obtained for a typical patient after administration of standard dosage regimens. PKPD simulations were performed using the PK model of ceftazidime from Sy et al. (43) and the PK model of CST from Kristoffersson et al. (44) assuming a typical patient (weight = 74.4 kg, creatinine clearance = 120 mL/min). The clinical dosing regimen of ceftazidime 2 g was administered every 8 h as a 2 h intravenous infusion and a loading dose of 9 million units (MIU) of colistin methanesulfonate sodium (CMS) followed by maintenance CMS doses (4.5 MIU) every 12 h were administered as 30 min intravenous infusions. The PK profiles of AVI were not simulated, and it was assumed that the AVI concentration was sufficient to inhibit beta-lactamases but not for its own bactericidal effect.

## RESULTS

### *In vitro* susceptibility testing

MICs of CZA and CST and the resistance genes of the four *K. pneumoniae* isolates are presented in Table 1. All the strains were susceptible to CZA [MICs ranging from 2 to 8 mg/L, EUCAST resistance breakpoint >8 mg/L (45)] and to CST [MICs ranging from 0.125 to 0.25 mg/L, EUCAST resistance breakpoint >2 mg/L (45)].

**TABLE 1:** Susceptibility to ceftazidime/avibactam and colistin of the *K. pneumoniae* isolates^*b*^

*K. pneumoniae* isolate	ST	Resistance genes^*a*^	CZA MIC (mg/L)	CST MIC (mg/L)
NARA1295	ST512	KPC-3, SHV-11 (35Q), TEM-1D, OmpK35 (25%), OmpK36 (GD)^*c*^	8	0.125
NARA1584	ST101	KPC-3, SHV-1, OmpK35 (17%), OmpK36 (TD)^*c*^	8	0.125
NARA1182	ST512	KPC-3, SHV-11 (35Q), OmpK35 (25%), OmpK36 (GD)^*c*^	4	0.125
NARA 864	ST348	KPC-3, SHV-11 (35Q), TEM-1D, OXA-9	2	0.25

^*a*^ Resistance genes were identified using Kleborate (35).

^*b*^ MIC: Minimum inhibitory concentration; CZA: ceftazidime/avibactam (fixed at 4 mg/L), EUCAST resistance breakpoint >8 mg/L; CST: colistin, EUCAST resistance breakpoint > 8 mg/L; CST: colistin, EUCAST resistance breakpoint > 2 mg/L.

^*c*^ GD: guanidine-aspartate insertion and TD: threonine-Aspartate insertion at position aa134-135.

### PKPD modeling of single drug and combination effects in TKC

**Fig 2:**
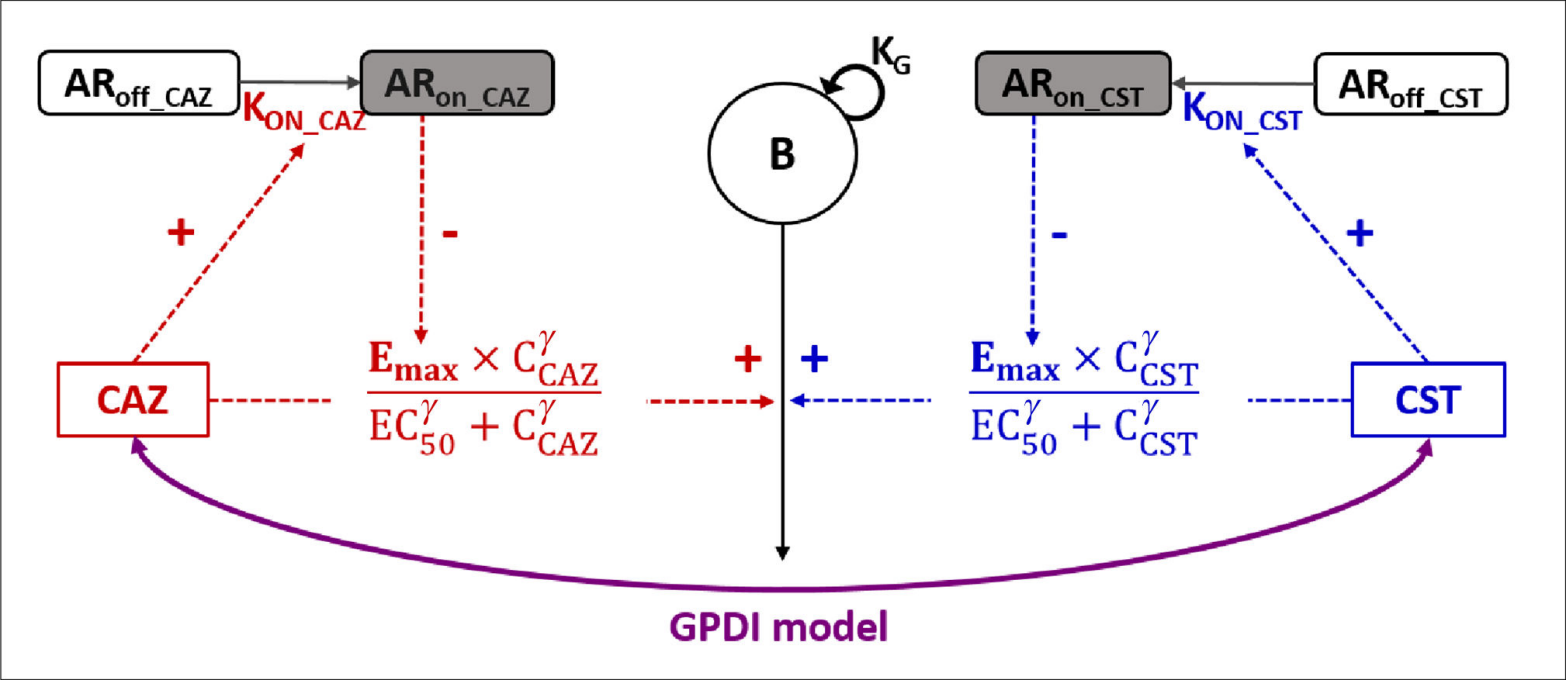
Pharmacodynamic model representation for time-kill experiments. *B*, total bacterial population; *K*_*G*_, growth rate; AR_off_ and AR_on_, states of adaptation; *K*_on_, adaptation rate. Emax, maximum bactericidal effect (reduced proportionally to AR_on_ until reaching a residual drug effect); EC50, concentration for which effect is 50% of Emax; *γ*, power parameter for effect. The GPDI model was implemented to describe the interaction between CZA and CST (see Materials and Methods) (42).

**Fig 3:**
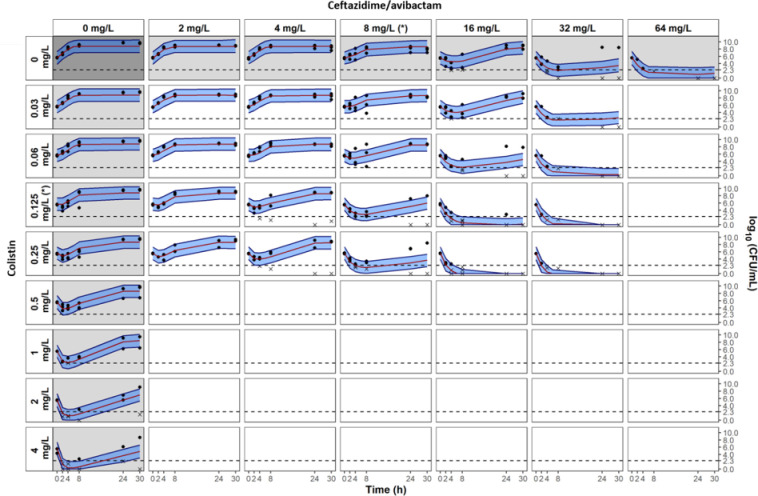
Visual predictive checks of the observed interaction model of time-kill experiments for *K. pneumoniae* NARA1295. Grey and white panels are associated to single drug and combination experiments, respectively. Measured CFU are represented by dots. For graphical representation, data below limit of quantification are represented by cross at their measured values. Median percentile from simulations with the observed interaction model is represented by red line and the 80% prediction interval between 10th and 90th percentiles is represented by the blue shaded areas. Limit of quantification is represented by the dashed line at 2.3 log_10_CFU/mL. MICs of CZA and CST are indicated by (*). Avibactam concentration was fixed at 4 mg/L.

A schematic representation of the model is shown in Fig. 2 and parameter estimates are given in Table 2. The fit between model predictions and observations is confirmed by the VPCs presented in Fig. 3 for the NARA1295 isolate and in Fig. S1–S3 for the other ones. The predictions’ vs observations’ plots are presented in Fig. S8 –S11.

**TABLE 2:** Full parameter estimates of the observed interaction models

		*K. pneumoniae* N1295	*K. pneumoniae* N1584	*K. pneumoniae* N1182	*K. pneumoniae* N864
Parameter	Description	Median (RSE%)^*b*^	Median (RSE%)	Median (RSE%)	Median (RSE%)
INOC	Estimated inoculum size (log_10_CFU/mL)	5.63 (2%)	5.62 (2%)	5.53 (2%)	5.68 (2%)
*K*_*G*_	Apparent growth rate constant (*h* ^−1^)	1.40 (13%)	1.77 (9%)	1.78 (13%)	1.84 (fixed)
Bmax	Maximal bacterial population supported by the system (log_10_CFU/mL)	8.80 (1%)	9.13 (1%)	8.43 (1%)	9.01 (2%)
Emax__CZA_	Maximum kill rate constant of CZA (*h* ^−1^)	4.59 (19%)	6.27 (14%)	7.51 (12%)	9.17 (16%)
EC_50_CZA_	CZA concentration required to achieve 50% of Emax__CZA_ (mg/L)	13.39 (12%)	13.47 (10%)	2.51 (12%)	0.46 (6%)
*γ*_CZA_	Sigmoidicity parameter for CZA effect	2.13 (16%)	1.84 (10%)	1.53 (11%)	1.79 (9%)
*K*_ON_CZA_	Rate constant for development of adaptive resistance to CZA (*h* ^−1^)	0.24 (51%)	0.32 (32%)	0.36 (25%)	0.66 (25%)
*K*_OFF_	Rate constant for reversal of adaptive resistance (*h* ^−1^)	0 (fixed)	0 (fixed)	0 (fixed)	0 (fixed)
ARmax__CZA_	Maximal reduction of Emax__CZA_ when bacteria are in the adapted state (%)	71.9 (8%)	73.0 (5%)	78.6 (4%)	82.5 (3%)
Emax__CST_	Maximum kill rate constant of CST (*h* ^−1^)	13.12 (41%)	8.78 (23%)	14.31 (50%)	8.81 (31%)
EC_50_CST_	CST concentration required to achieve 50% of Emax__CST_ (mg/L)	0.60 (46%)	0.33 (15%)	0.33 (19%)	0.21 (11%)
*γ*_CST_	Sigmoidicity parameter for CST effect	0.96 (17%)	1.13 (11%)	1.21 (12%)	1.16 (6%)
*K*_ON_CST_	Rate constant for development of adaptive resistance to CST (*h* ^−1^)	0.65 (56%)	0.52 (39%)	1.18 (61%)	0.75 (47%)
ARmax__CST_	Maximal reduction of Emax__CST_ when bacteria are in the adapted state (%)	91.8 (3%)	83.1 (4%)	87.0 (6%)	80.3 (6%)
Perpetrator	Drug responsible for the interaction	CST	CST	CST	CST
INT_EC_50_ ^*a*^	Maximal fractional change of the EC50 of the victim drug (%)	−51.4 (10%)	−48.3 (10%)	−48.6 (12%)	−
EC_50_INT_EC50_	Concentration of the perpetrator drug required to achieve 50% of INT_EC_50_ (mg/L)	0.06 (5%)	0.06 (4%)	0.07 (17%)	−
*γ*_INT_EC50_	Sigmoidicity parameter for the combination effect	10 (fixed)	10 (fixed)	10 (fixed)	−
INT_Emax^*a*^	Maximal fractional change of the Emax of the victim drug (%)	−	−	−	−65.8 (16%)
EC_50_INT_Emax_	Concentration of the perpetrator drug required to achieve 50% of INT_Emax (mg/L)	−	−	−	0.39 (fixed)
*γ*_INT_Emax_	Sigmoidicity parameter for the combination effect	−	−	−	1 (fixed)
RES_ADD	Additive residual error (log_10_CFU/mL)	1.74 (7%)	1.01 (7%)	1.44 (7%)	2.25 (6%)

^*a*^ (−) indicates decrease of the parameter and (+) indicates an increase of the parameter due to the interaction.

^*b*^ Relative standard error.

For each isolate, after an initial decay (typically faster with CST than with CZA), regrowth was observed with drug alone at concentrations equal to their respective MICs. Depending on the strain, the concentrations of CZA or CST required to avoid the emergence of resistance were 4–8× the MIC and 16–32× the MIC, respectively. In combination, concentrations of both drugs in the range of 1–2× the MIC systematically prevented the emergence of resistance. Concentrations of 2–4× the MIC of CZA combined with low CST concentrations from 0.25 to 1× the MIC were also able to prevent the emergence of resistance. Of note, these results were observed at concentrations clinically achievable.

### Evaluation of the pharmacodynamic interaction in TKC

According to the GPDI model, CST decreased the EC_50_ of CZA by approximately 50% at maximum in the NARA1295, NARA1584, and NARA1182 isolates (Table 2). In the *K. pneumoniae* NARA864, CST reduced the Emax of CZA by 65% at maximum (Table 2).

**Fig 4:**
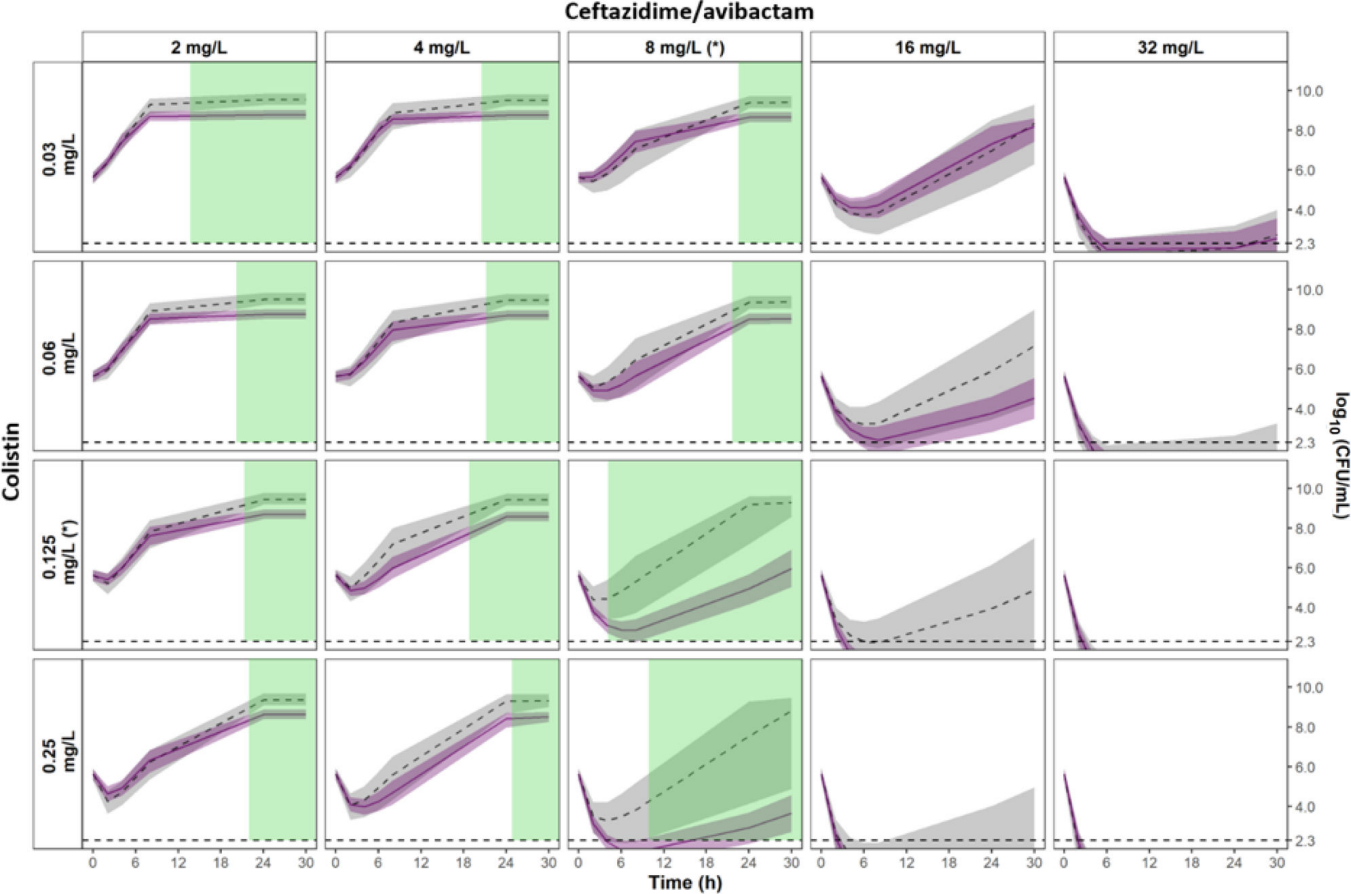
Comparison of the 95%CIs of the observed interaction vs the expected additivity for *K. pneumoniae* NARA1295. The 95%CI of the expected additivity, obtained by parametric bootstrap, is represented by grey areas (*n* = 980 runs) and the corresponding median percentile is represented by the dashed line. The 95%CI of the observed interaction is represented by light purple areas and the median percentile is represented by the solid line (*n* = 976 runs). Statistically significant areas of synergy (nonoverlapping 95%CI) are highlighted in green. The limit of quantification is represented by the horizontal dashed line at 2.3 log_10_CFU/mL. MICs of CZA and CST are indicated by (*).

**Fig 5:**
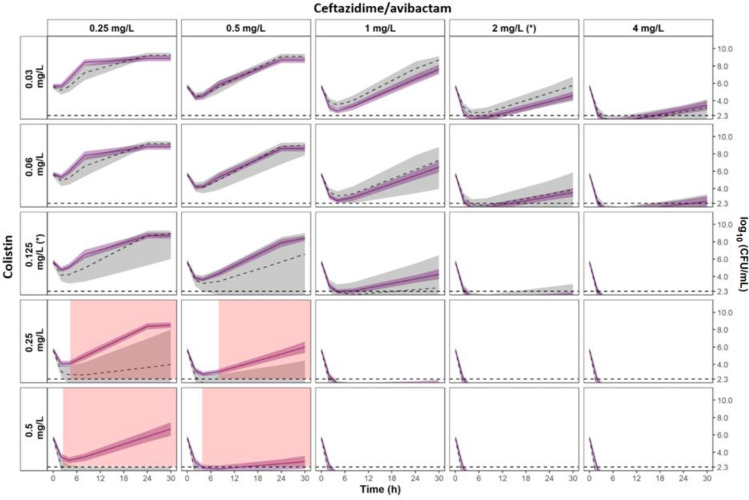
Comparison of the 95%CIs of the observed interaction vs the expected additivity for *K. pneumoniae* NARA864. The 95%CI of the expected additivity, obtained by parametric bootstrap, is represented by grey areas (*n* = 1,000 runs) and the corresponding median percentile is represented by the dashed line. The 95%CI of the observed interaction is represented by light purple areas and the median percentile is represented by the solid line (*n* = 1,000 runs). Statistically significant areas of antagonism (nonoverlapping 95%CI) are highlighted in red. The limit of quantification is represented by the horizontal dashed line at 2.3 log_10_CFU/mL. MICs of CZA and CST are indicated by (*).

Significant synergistic effects were observed at late times for the NARA1295 isolate at CZA concentrations lower than 8 mg/L (highlighted in green in Fig. 4). On the contrary, significant antagonistic effects were observed for the NARA864 at CZA concentrations of 0.25–0.5 mg/L and CST at 0.25–0.5 mg/L (highlighted in red in Fig. 5). For both NARA1584 and NARA1182, the 95%CI of the expected additivity and the 95%CI of the observed interaction model overlapped widely, indicating additive effects (Fig. S4 and S5). It should be noted that for those strains, the 95%CI of the expected additivity was wide and limited the power of the statistical comparison.

### Simulation of the combination effect at clinical concentrations

**Fig 6:**
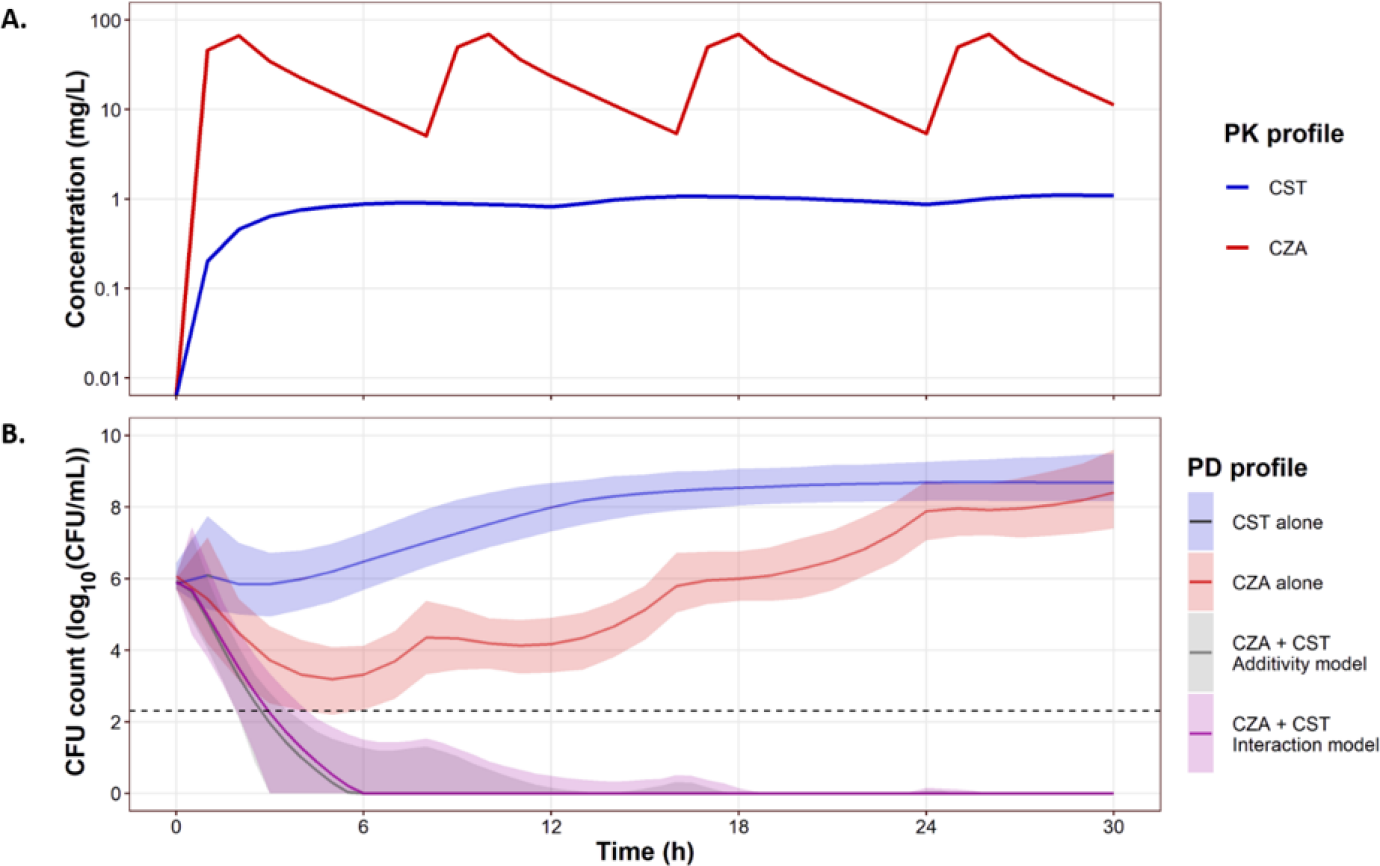
Simulation of expected *in vitro* effects (lower panel) in combination for typical concentration profiles (upper panel) obtained in patients after standard CZA (2/0.5 g q8h as 2 h infusion) and CST (9 MIU CMS + 4.5 MIU CMS q12h as 30 min infusion) doses.

Simulation results for the *K. pneumoniae* NARA1295 are presented in Fig. 6. The usual dosing regimen of CST administered alone was predicted to be ineffective, meanwhile CZA alone produced early killing of the bacteria followed by regrowth (Fig. 6B). By combining both dosing regimens, the simulations predicted a complete eradication of the bacteria without regrowth. Moreover, simulations performed using the additivity assumption of the observed effects for antibiotics alone and simulations performed using the interaction model built from the observed effects in combinations were very close, with 95%CIs systematically overlapping (Fig. 6B). This meant that for varying concentrations such as those observed under clinical conditions, our *in vitro* data suggested that the PD interaction between CZA and CST should be additive.

Since the combined effect of the usual CZA and CST dosages was very strong, alternative dosing regimens were simulated. Depending on the type of administration (intermittent or continuous infusion) and the simulated doses (to achieve the targeted average concentrations), the interaction could be additive or synergistic (Fig. S6 and S7, simulated with large data set to have a high statistical power).

## DISCUSSION

Various methods for characterizing PD interactions between antibiotics can be found in the scientific literature. The most frequent is the checkerboard method with turbidity readout at 24 h and calculation of FICI. If we had interpreted our experimental time-kill results as checkerboards, i.e.*,* considering only the measurements at 24 h and a visible turbidity for bacterial counts higher than 10^7^ CFU/mL, the combination would have been additive in the NARA1295 (FICI = 0.5), NARA1182 (FICI = 0.5), and NARA864 (FICI = 0.8) and synergistic in the NARA1584 (FICI = 0.4). Few articles in the literature characterized *in vitro* antibiotic PD interactions from time-kill experiments using semi-mechanistic PKPD modeling (21). In these cases, the comparison with different additivity models (Bliss, Loewe, or other) was mathematically done by evaluating the improvement of the likelihood of the PKPD model when adding an interaction parameter. Considering only the statistical comparison of the interaction parameter, the effect would have been considered synergistic in three isolates (NARA1295, NARA1584, and NARA1182) and antagonistic in the remaining one (NARA864). However, interpreting a change in a PD parameter in combination may be complicated while the CFU profiles over time can highlight how this change impacts the effect. This was previously done by Chen et al. (46) to characterize the interaction between anti-tuberculosis drugs, based on comparison of effects. They graphically compared the typical CFU profiles predicted under the expected additivity assumption to the typical profiles observed in combination. Yet, this “graphical” approach lacked a criterion for classifying the interaction as antagonistic, additive, or synergistic. We have estimated the uncertainty on the model parameters in order to construct the IC95% of the observed and predicted CFU profiles under an assumption of additivity of effects (according to Bliss). Comparing the IC95% thus enables us to statistically define the type of interaction at different times and concentrations. Overall, the results showed that, in the range of tested concentrations, the effects of CZA and CST were additive over time. However, looking in more detail synergistic effects were observed under specific conditions for NARA1295 and NARA1584 (Fig. 4 and Fig. S4, respectively) and antagonistic effects were observed when high concentrations of CST and low concentrations of CZA were associated for NARA864 (Fig. 5).

A limitation of these results was that the type of interaction varied with antibiotic concentrations and time, so it was difficult to conclude globally for the combination studied. Of primary interest to clinicians is whether or not the combination will be effective in a clinical setting, i.e., with varying concentrations over time. This is why the expected *in vitro* effects for antibiotic concentrations typically observed in patients were simulated using PK models from the literature and the PD model developed with our experimental data. These simulations showed that the CZA + CST combination, used at usual dosing regimens against these four isolates, should be additive. It is also interesting to note that our simulations showed that the type of interaction could depend on the dosage regimen. Indeed, the type of interaction varied according to the concentration range and time, and thus we were able to determine some dosage regimens for which a synergistic effect was predicted, while for others an additive effect was expected (Fig. S6 and S7). The synergistic nature of the pharmacodynamic interaction is not necessarily useful for clinical use if the molecules are active. In our case, there was no need to look for a synergistic dosing regimen since our results suggested that the usual regimens should be effective, but one can imagine scenarios where dosing regimens could be optimized by seeking synergistic rather than additive (or antagonistic) interactions. Please note that our simulations had an illustrative purpose, and that we made the choice to simulate typical PK profiles from the literature and applying them to particular patient populations. Another choice of simulation PK model might have led to different results. An application of our results to a particular indication in a special patient population would require the application of an adapted PK model. Also, it should be noted that instead of doing simulations based on the PD model obtained from time-kill experiments with constant antibiotic concentrations, it would have been preferable to perform experiments with variable concentrations (hollow-fiber system), or even from *in vivo* infection models, but these are much more labor intensive.

The predictive power of our model is naturally limited by the fact that it was *in vitro* and that the experiments involved only four bacterial strains. TKC experiments, especially in combination, are laborious to carry out and it was difficult to study more strains. We carried out dynamic checkerboard experiments with CFU assessment at 24 h on nine *K. pneumoniae* strains, showing that the interaction, based on the GPDI model, was synergistic for four strains and additive for the remaining ones (Table S1). This reinforces the predictive character of the model we have developed, while remaining limited by its *in vitro* nature.

In order to construct the confidence intervals for comparing the profiles predicted under the expected additivity and the profiles obtained experimentally in combination, we performed a parametric bootstrap. Other methods for estimating parameter uncertainty were possible, and the variance-covariance matrix, computed during the $COVARIANCE step in NONMEM, was the fastest and easiest way to assess parameter uncertainty. However, the corresponding 95%CIs were quite large, and this method being not very robust under certain conditions (35), we tested other methods. A non-parametric bootstrap (with resampling in the original data set) was not appropriate because stratification by concentration level would have led to an insufficient number of data per condition and to a resampling equivalent to the initial data set. We, therefore, performed a parametric bootstrap, and the estimated 95%CIs were significantly narrower than those obtained with the NONMEM variance-covariance matrix. As a comparison, we also performed a Sampling Importance Resampling (SIR) procedure (35, 47), using the covariance matrix from NONMEM $COV step as proposal distribution, and the results obtained were close to those of the parametric bootstrap (data not shown). There are, therefore, several possibilities for estimating the uncertainties of the model parameters and it is important to choose one that is adapted to the data. This choice will affect the size of the confidence intervals and, therefore the conclusion regarding the type of interaction.

The power of the statistical comparison of the predicted effects, i.e.*,* the probability of concluding to a significant difference from the predicted effects under the expected additivity model, depended on the width of the confidence intervals. In order to reduce the 95%CI of the expected additivity and increase the discriminating power of this analysis, it is necessary to limit the uncertainty of the PD model. This can be done by gathering more single drug data, or by adapting the structure of the PKPD model to each antibiotic/strain pair. In order to facilitate inter-strain comparisons, we chose the same PD model for the four isolates, but using different models we could have reduced the parameters uncertainty and thus reduced the 95%CI. It would also have been possible to develop a model incorporating variability in one or more parameters. However, it was difficult to identify on which parameter to add this variability and at what level this variability applied (between tubes in the same experiment or between experiments). It was, therefore, decided not to estimate any variability other than the residual error. Also, optimal design methodology can be used to develop the most informative experiment (21).

The PD interactions were characterized by comparison to the expected additivity defined by Bliss independence. We made this choice based on the assumption that CZA and CST effects were independent. However, we could have used another definition for classifying the PD interactions and the proposed method is applicable with other definitions such as Loewe additivity. The effect of the combination could also be compared to the most active single agent or to a pharmacologically relevant threshold (e.g., a decrease in CFU counts ≥2-log10 compared to the most active single agent). Furthermore, our approach could also be applied to pharmacodynamic models with varied structures, including those with resistant bacterial subpopulations.

The CZA + CST combination was little studied *in vitro*. Shields et al. characterized the interaction between CZA and CST on carbapenem-resistant enterobacterales (including KPC-producing *K. pneumoniae*) using TKCs but at a single CST concentration (2 mg/L) (11). They defined the synergy as a decrease in CFU counts at 24 h higher than 2-log_10_ compared to the most active single agent and the antagonism as an increase in CFU counts at 24 h higher than 1-log_10_ compared to the most active single agent and found synergy for 13% (3/24) of the strains, additivity for 41% (10/24) of the strains, and antagonism for 46% (11/24) of the strains. Yet, it is undesirable to use only the CFU counts at 24 h since TKCs also provide information on the bacterial regrowth over time. Of note, none of the regrowth observed at 24 h in the presence of one or both antibiotics was systematically related to gene mutations. Our approach showed that on our strains, the CZA + CST interaction was mainly additive and that synergistic or antagonistic effects could be observed at certain times and for specific concentration levels. Moreover, our results demonstrated that the clinical dosing regimens of both drugs administered in combination prevented the emergence of resistance against all the *K. pneumoniae* isolates, whereas bacterial regrowth was observed with drugs given alone, particularly in those with MICs close to the breakpoint. Overall, these results support the potential of the CZA + CST combination.

We acknowledge that TKCs were performed using a fixed avibactam concentration (4 mg/L). In the equations reported in this article, the term “CZA” characterizes the concentration of ceftazidime and it should be understood that this term corresponds to the concentration of ceftazidime associated with 4 mg/L of avibactam. Moreover, in our PD model, it was assumed that the AVI concentration was sufficient to inhibit beta-lactamases but not for its own bactericidal effect. However, previous studies have reported that avibactam could also potentiate ceftazidime effect and induce direct bacterial killing at higher concentrations (48, 49). The PK of avibactam was not simulated since our *in vitro* data could not support the activity of avibactam at varying concentrations. Hence, bacterial profiles simulated for ceftazidime/avibactam alone and in combination with colistin must be considered with caution regarding model assumptions.

In conclusion, our results showed that the type of interaction varied as a function of concentrations and time. The checkerboard methodology, which evaluates the interaction at a given time, is, therefore, insufficient. Time-kill curves, associated with PKPD modeling, can help to answer this question, particularly to characterize the pharmacodynamic interaction that is expected in clinical practice. Against these four MDR *K. pneumoniae* isolates, the bactericidal effect of CZA + CST combination was predicted to be additive at clinical concentrations and to prevent the emergence of resistance.

## SUPPLEMENTAL MATERIAL

Figures S1 to S11, Table S1, Code S1, Code S2 aac.00301-23-s0001.docx Supplemental include: -Additional figures of the analysis -PD interaction results in 9 *K. pneumoniae* strains -Codes of the NONMEM control files.

ASM does not own the copyrights to Supplemental Material that may be linked to, or accessed through, an article. The authors have granted ASM a non-exclusive, world-wide license to publish the Supplemental Material files. Please contact the corresponding author directly for reuse.
