# Supplementary material for: An improved PKPD modeling approach to characterize the pharmacodynamic interaction over time between ceftazidime/avibactam and colistin from in vitro time-kill experiments against multidrug-resistant Klebsiella pneumoniae isolates
Source: Antimicrob Agents Chemother. 2023 Sep 8;67(10):e00301-23. doi: 10.1128/aac.00301-23 (PMC10583682; doi:10.1128/aac.00301-23)
Supplement: Figures S1 to S11, Table S1, Code S1, Code S2 — Supplemental include: -Additional figures of the analysis -PD interaction results in 9 K. pneumoniae strains -Codes of the NONMEM control files. [file aac.00301-23-s0001.docx]

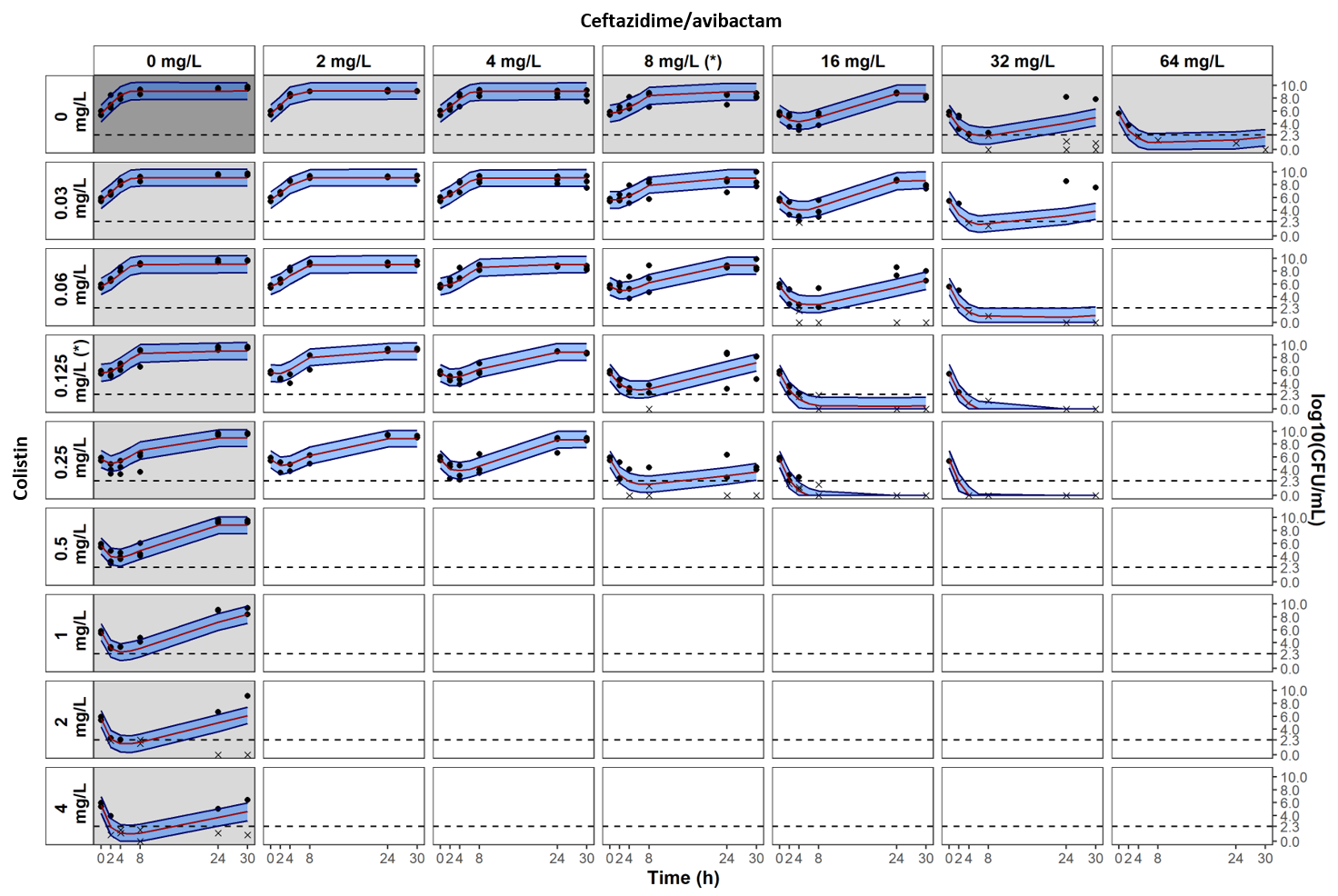


**Figure S1. Visual Predictive Checks of the observed interaction model for *K. pneumoniae* NARA1584 isolate**

Grey and white panels are associated to single drug and combination experiments, respectively. Measured CFU are represented by dots. For graphical representation, data below limit of quantification are represented by cross at their measured values. Median percentile from simulations with the observed interaction model is represented by red line and the 80% prediction interval between 10^th^ and 90^th^ percentiles is represented by the blue shaded areas. Limit of quantification is represented by the dashed line at 2.3 log_10_CFU/mL. MICs are indicated by (*). Avibactam concentration was fixed at 4 mg/L.


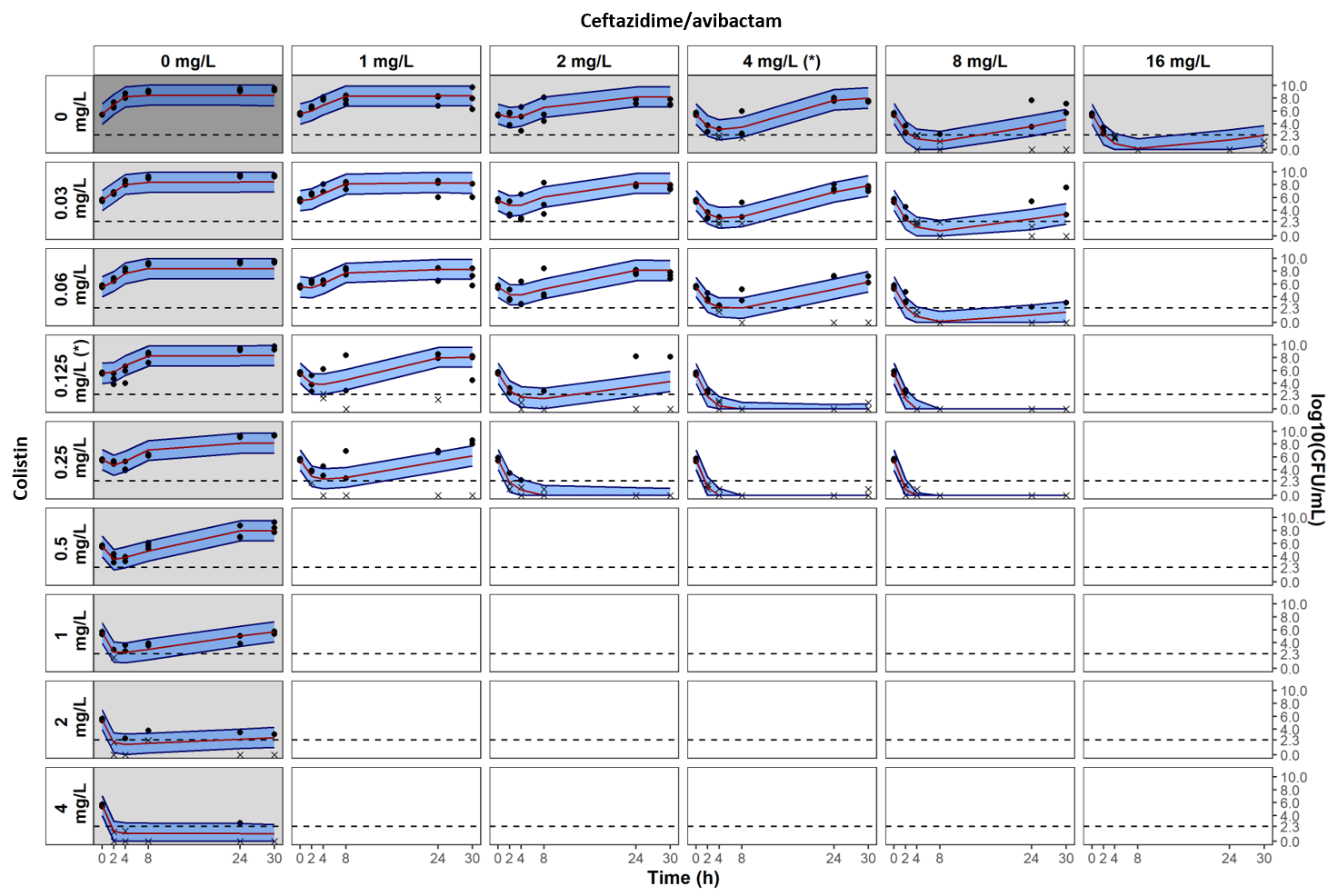


**Figure S2. Visual Predictive Checks of the observed interaction model for *K. pneumoniae* NARA1182 isolate**

Grey and white panels are associated to single drug and combination experiments, respectively. Measured CFU are represented by dots. For graphical representation, data below limit of quantification are represented by cross at their measured values. Median percentile from simulations with the observed interaction model is represented by red line and the 80% prediction interval between 10^th^ and 90^th^ percentiles is represented by the blue shaded areas. Limit of quantification is represented by the dashed line at 2.3 log_10_CFU/mL. MICs are indicated by (*). Avibactam concentration was fixed at 4 mg/L


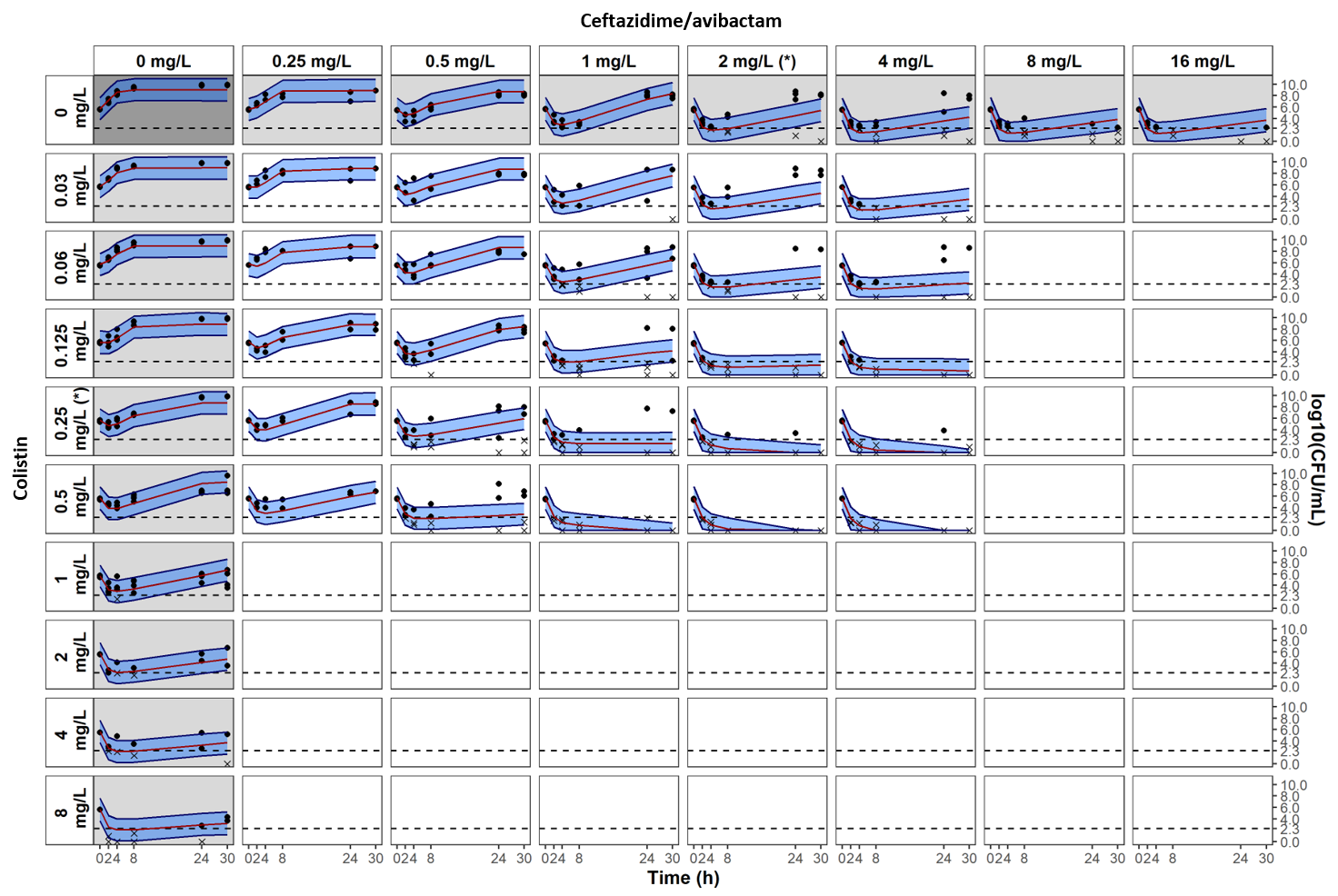


**Figure S3. Visual Predictive Checks of the observed interaction model for *K. pneumoniae* NARA864 isolate**

Grey and white panels are associated to single drug and combination experiments, respectively. Measured CFU are represented by dots. For graphical representation, data below limit of quantification are represented by cross at their measured values. Median percentile from simulations with the observed interaction model is represented by red line and the 80% prediction interval between 10^th^ and 90^th^ percentiles is represented by the blue shaded areas. Limit of quantification is represented by the dashed line at 2.3 log_10_CFU/mL. MICs are indicated by (*). Avibactam concentration was fixed at 4 mg/L.


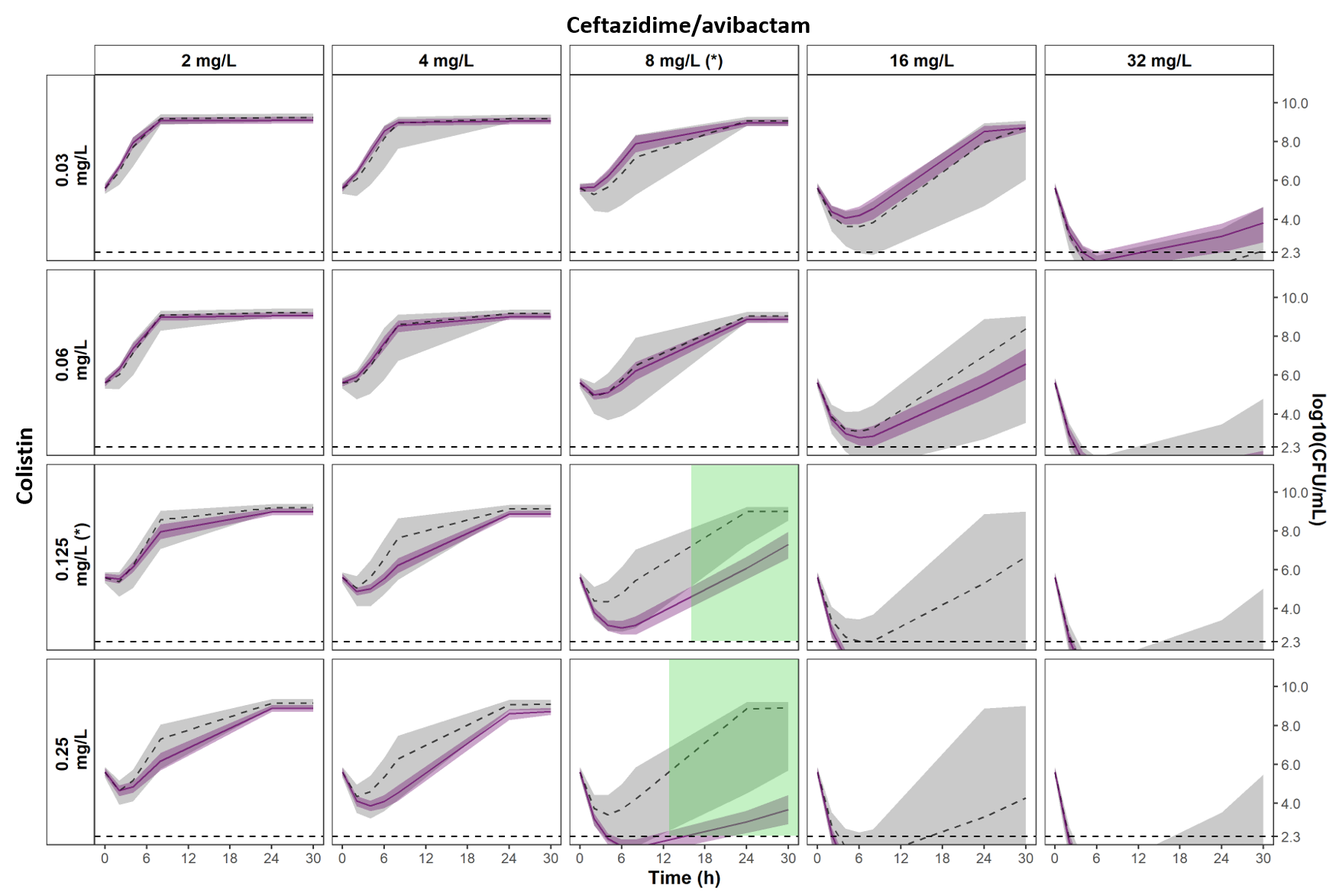


**Figure S4. Comparison of the 95%CIs of the observed interaction versus the expected additivity for *K. pneumoniae* NARA1584**

The 95%CI of the expected additivity, obtained by parametric bootstrap, is represented by grey areas (n=988 runs) and the corresponding median percentile is represented by the dashed line. The 95%CI of the observed interaction is represented by light purple areas and the median percentile is represented by the solid line (n=1000 runs). Statistically significant areas of synergy (non-overlapping IC95%) are highlighted in green. The limit of quantification is represented by the horizontal dashed line at 2.3 log_10_CFU/mL. MICs of CZA and CST are indicated by (*).


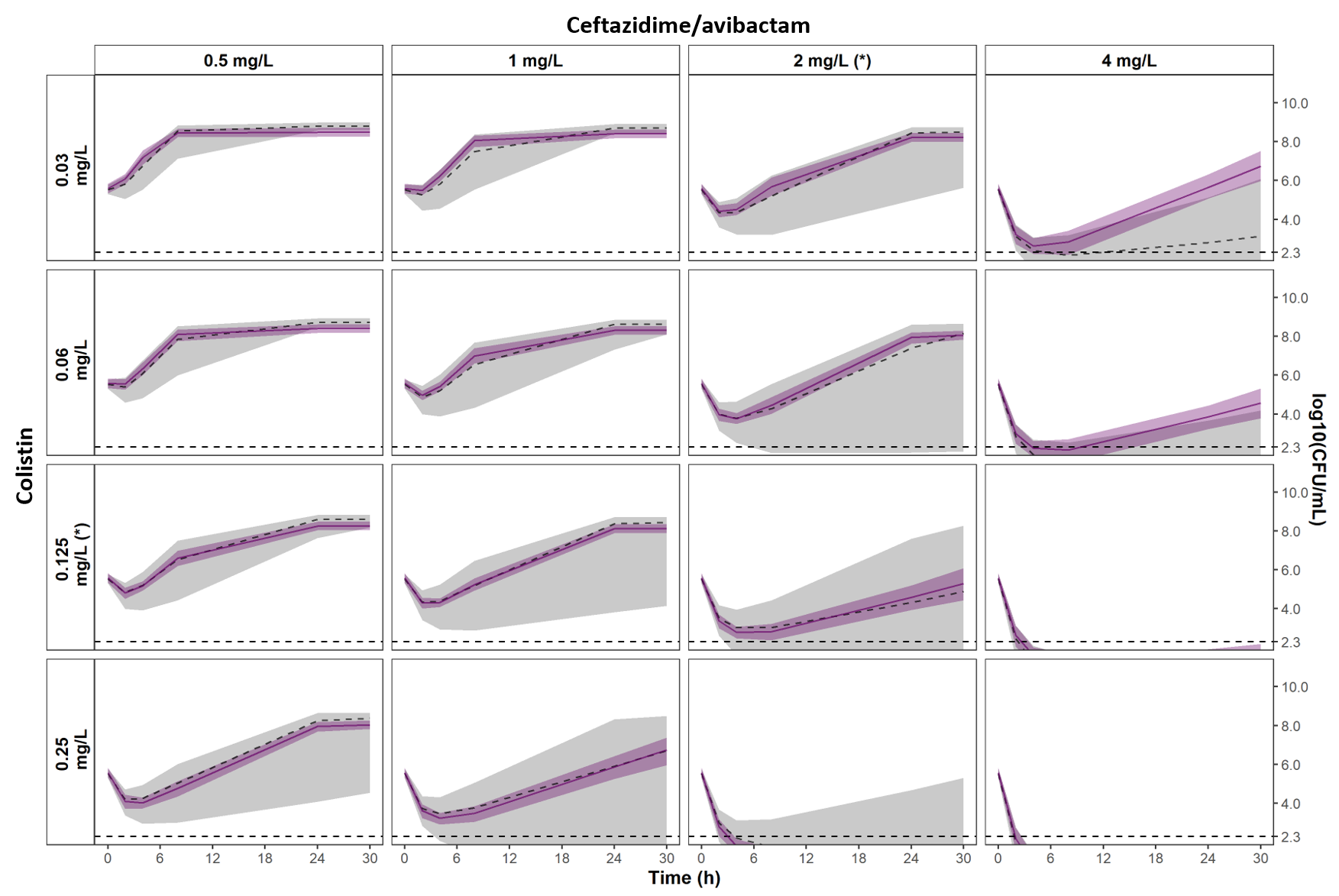


**Figure S5. Comparison of the 95%CIs of the observed interaction versus the expected additivity for *K. pneumoniae* NARA1182**

The 95%CI of the expected additivity, obtained by parametric bootstrap, is represented by grey areas (n=992 runs) and the corresponding median percentile is represented by the dashed line. The 95%CI of the observed interaction is represented by light purple areas and the median percentile is represented by the solid line (n=930 runs). The limit of quantification is represented by the horizontal dashed line at 2.3 log_10_CFU/mL. MICs of CZA and CST are indicated by (*).

**
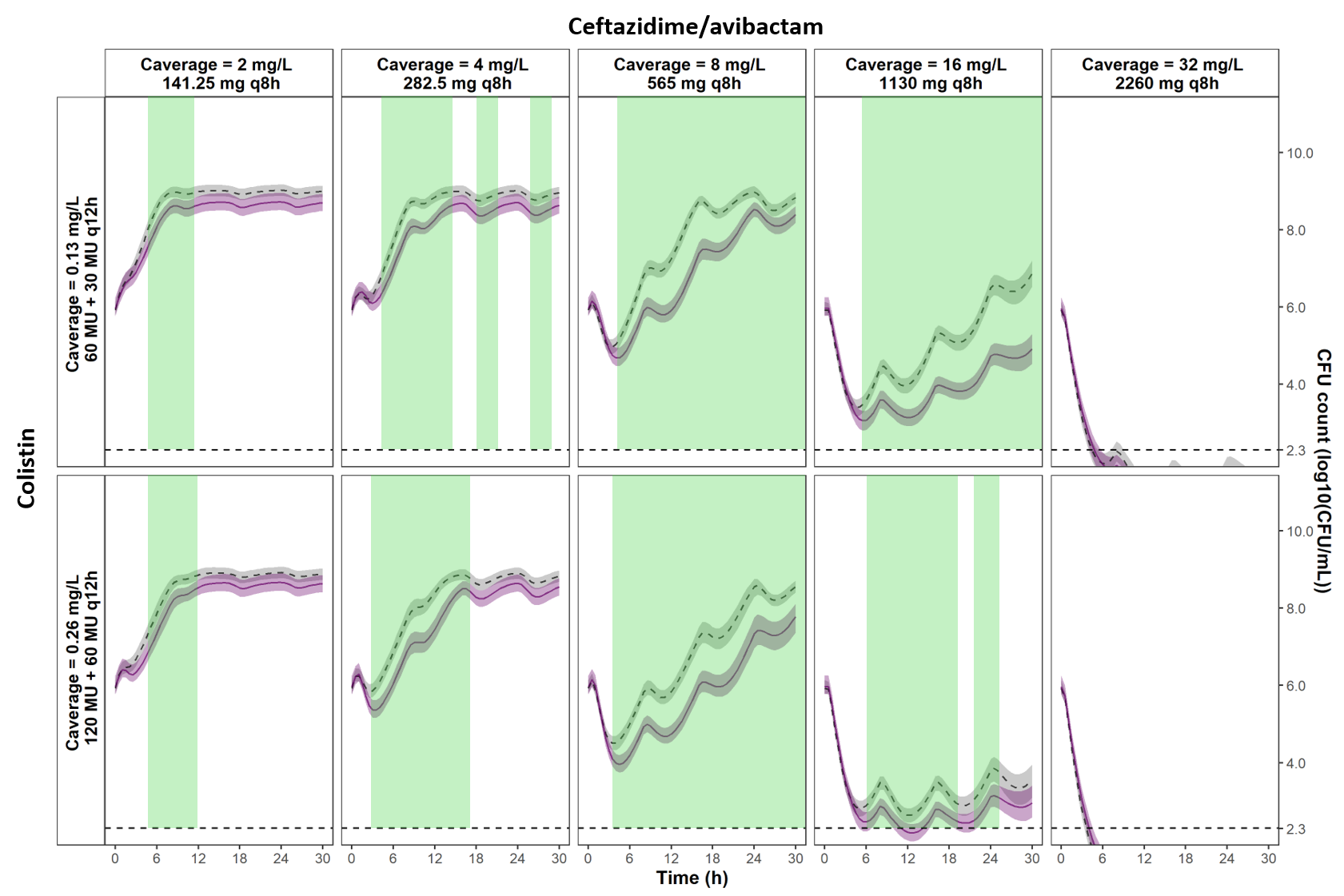
**

**Figure S6. Simulations of the combination effects after CZA and CST intermittent infusions**

The 95%CI of the expected additivity, obtained by parametric bootstrap, is represented by grey areas (n=1000 runs) and the median percentile is represented by the dashed line. The 95%CI of the observed interaction is represented by light purple areas (n=1000 runs) and the median percentile is represented by the solid line. Significant differences between both 95%CIs are highlighted in green and indicate synergy. The limit of quantification is represented by the horizontal dashed line at 2.3 log_10_CFU/mL. In order to obtain sufficiently narrow intervals and have a high statistical power, PK and PD profiles were simulated every 30 minutes for 30h.


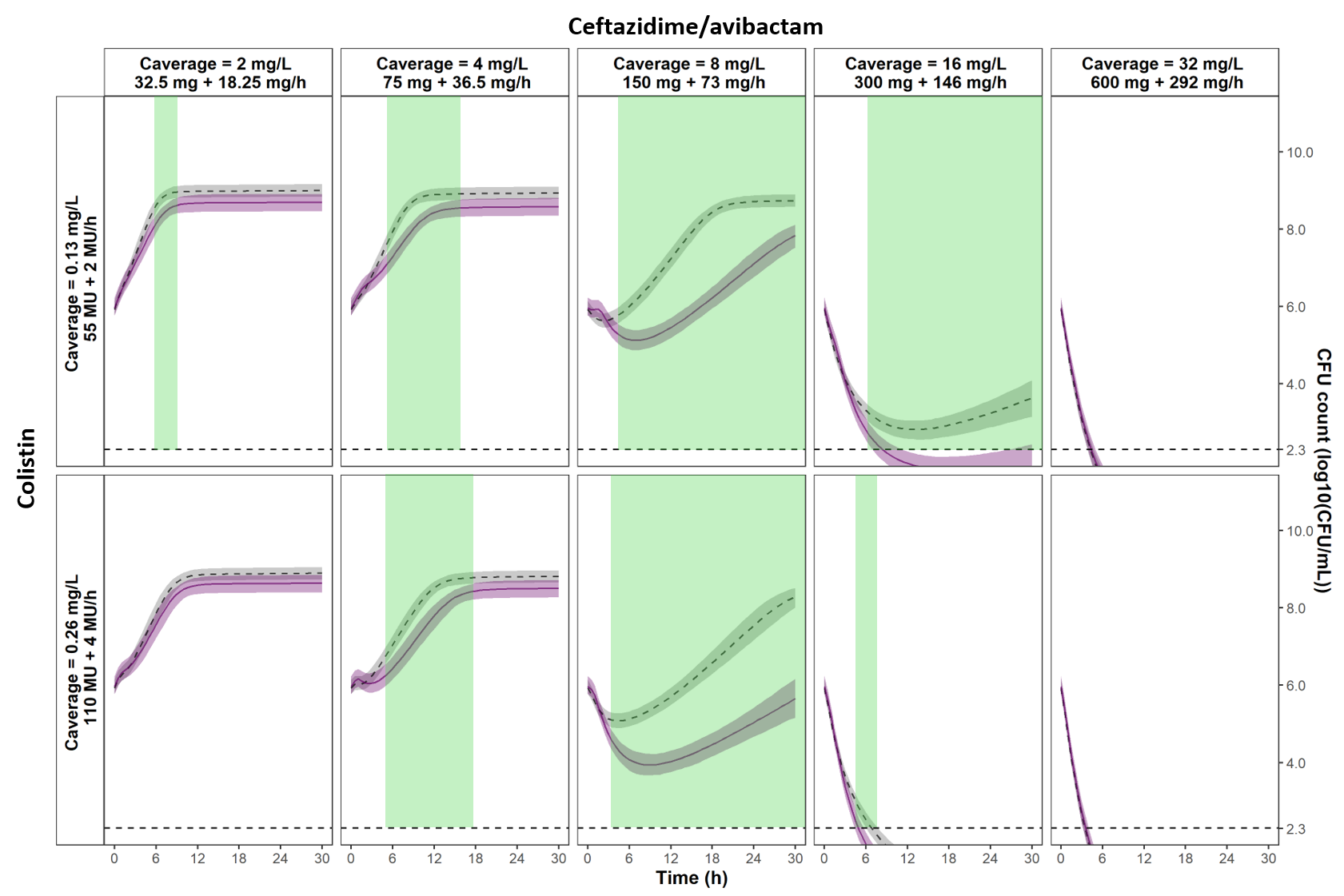

**Figure S7. Simulations of the combination effects after CZA and CST loading doses followed by continuous infusion**

The 95%CI of the expected additivity, obtained by parametric bootstrap, is represented by grey areas (n=1000 runs) and the median percentile is represented by the dashed line. The 95%CI of the observed interaction is represented by light purple areas (n=1000 runs) and the median percentile is represented by the solid line. Significant differences between both 95%CIs are highlighted in green and indicate synergy. The limit of quantification is represented by the horizontal dashed line at 2.3 log_10_CFU/mL. In order to obtain sufficiently narrow intervals and have a high statistical power, PK and PD profiles were simulated every 30 minutes for 30h.

**
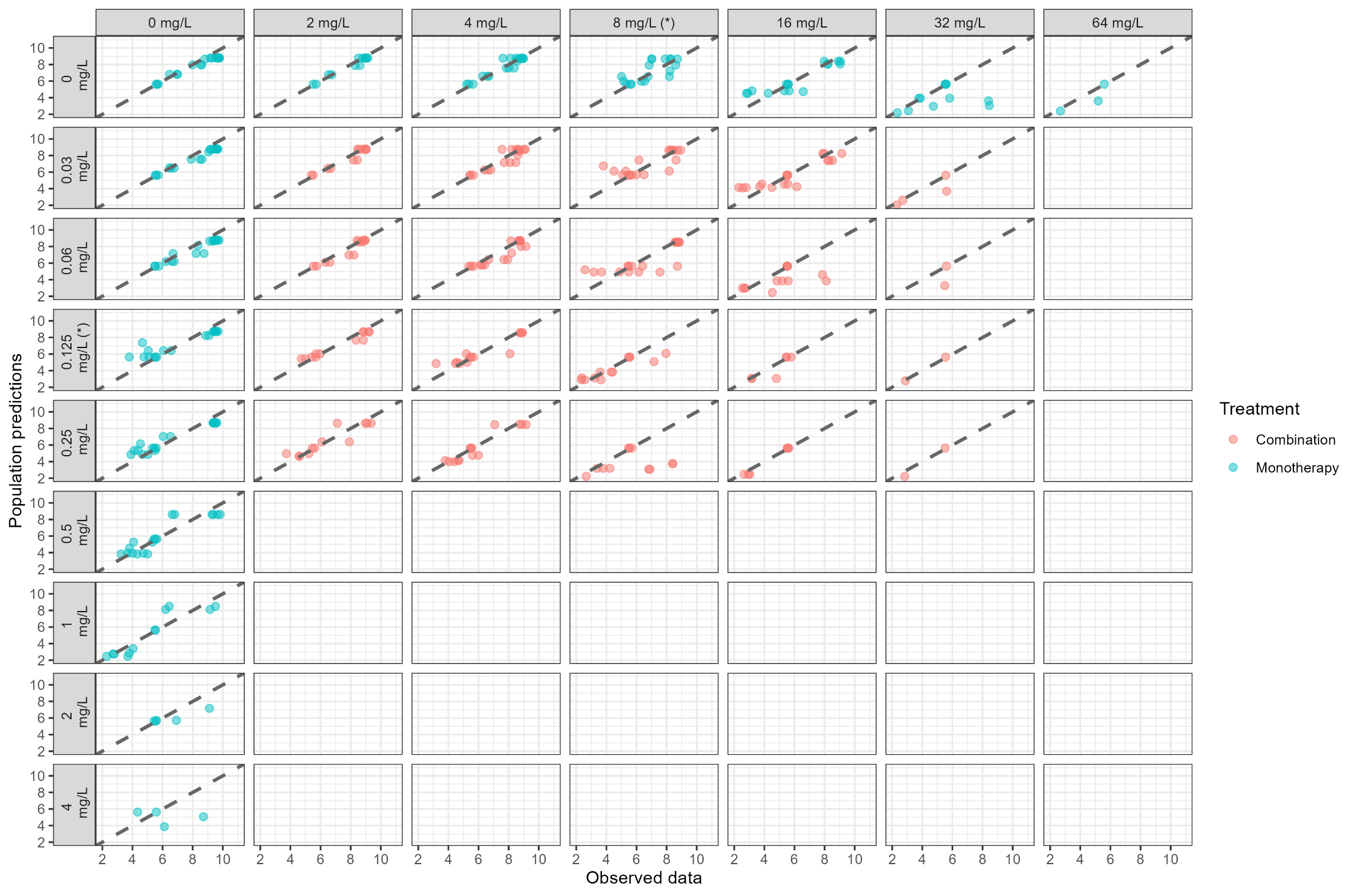

Figure S8. Observations vs predictions stratified by concentrations for *K. pneumoniae* NARA1295 isolate**

**
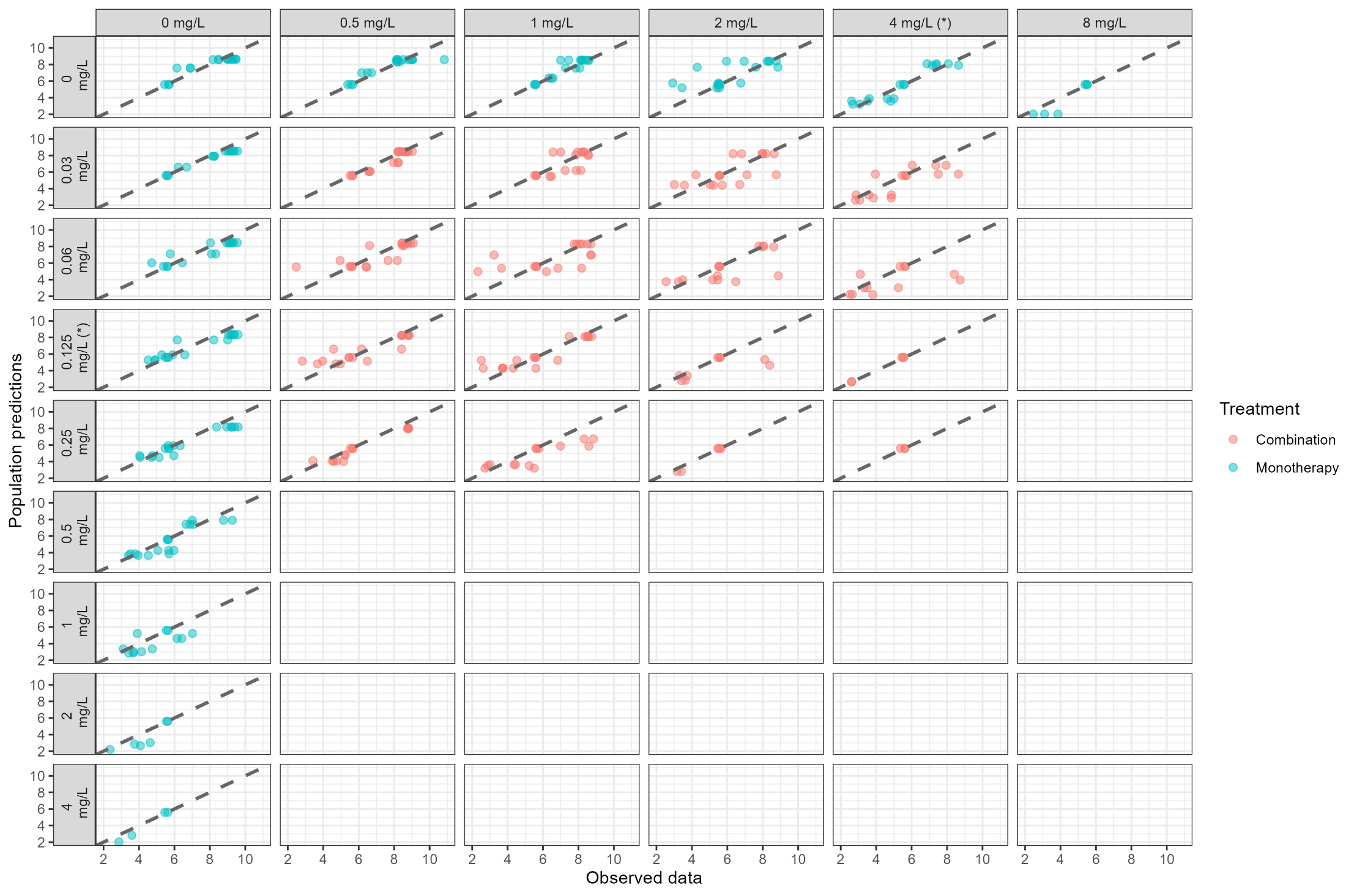

Figure S9. Observations vs predictions stratified by concentrations for *K. pneumoniae* NARA1182 isolate**

**
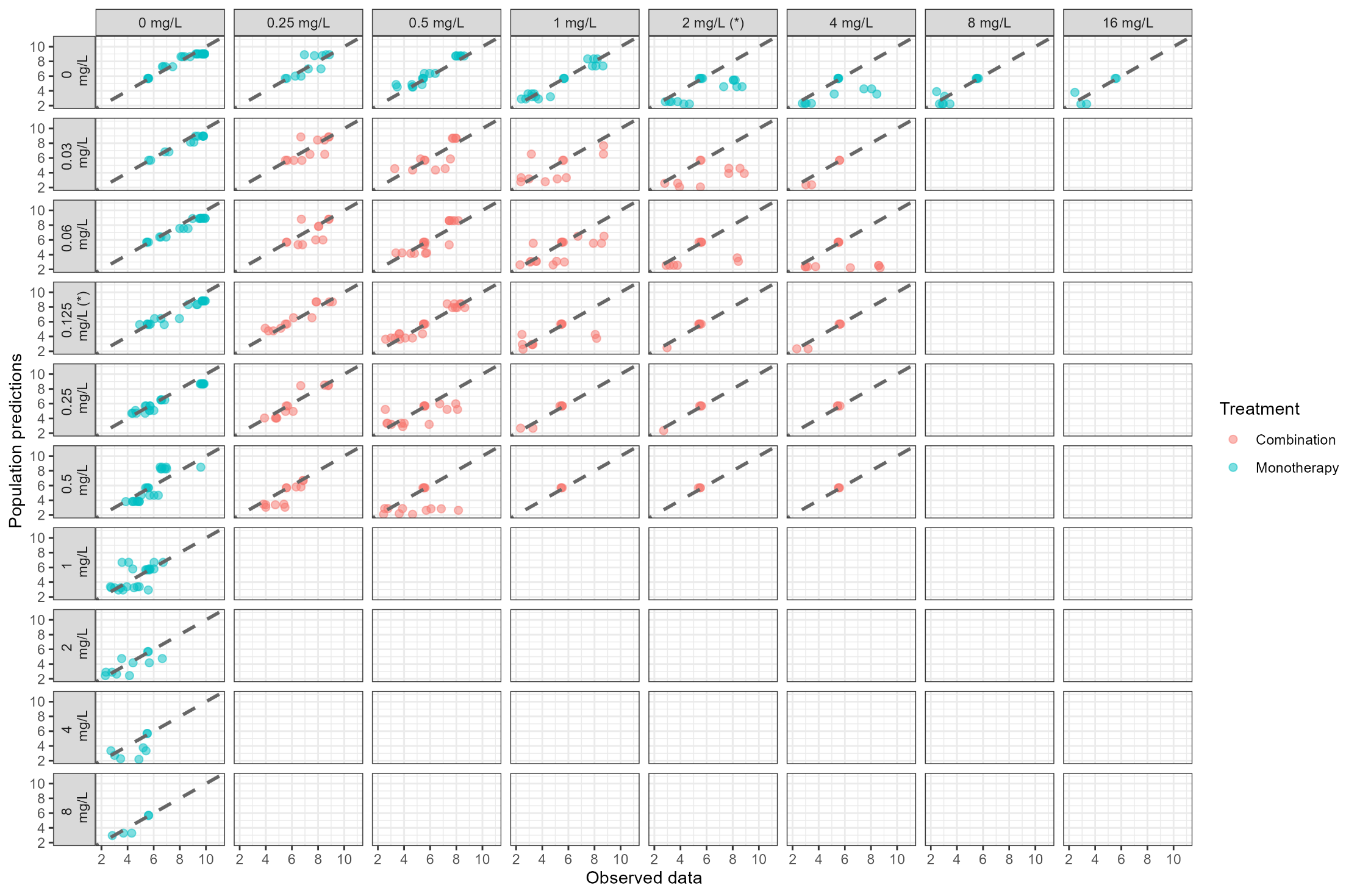
**

**Figure S10. Observations vs predictions stratified by concentrations for *K. pneumoniae* NARA864 isolate**

**
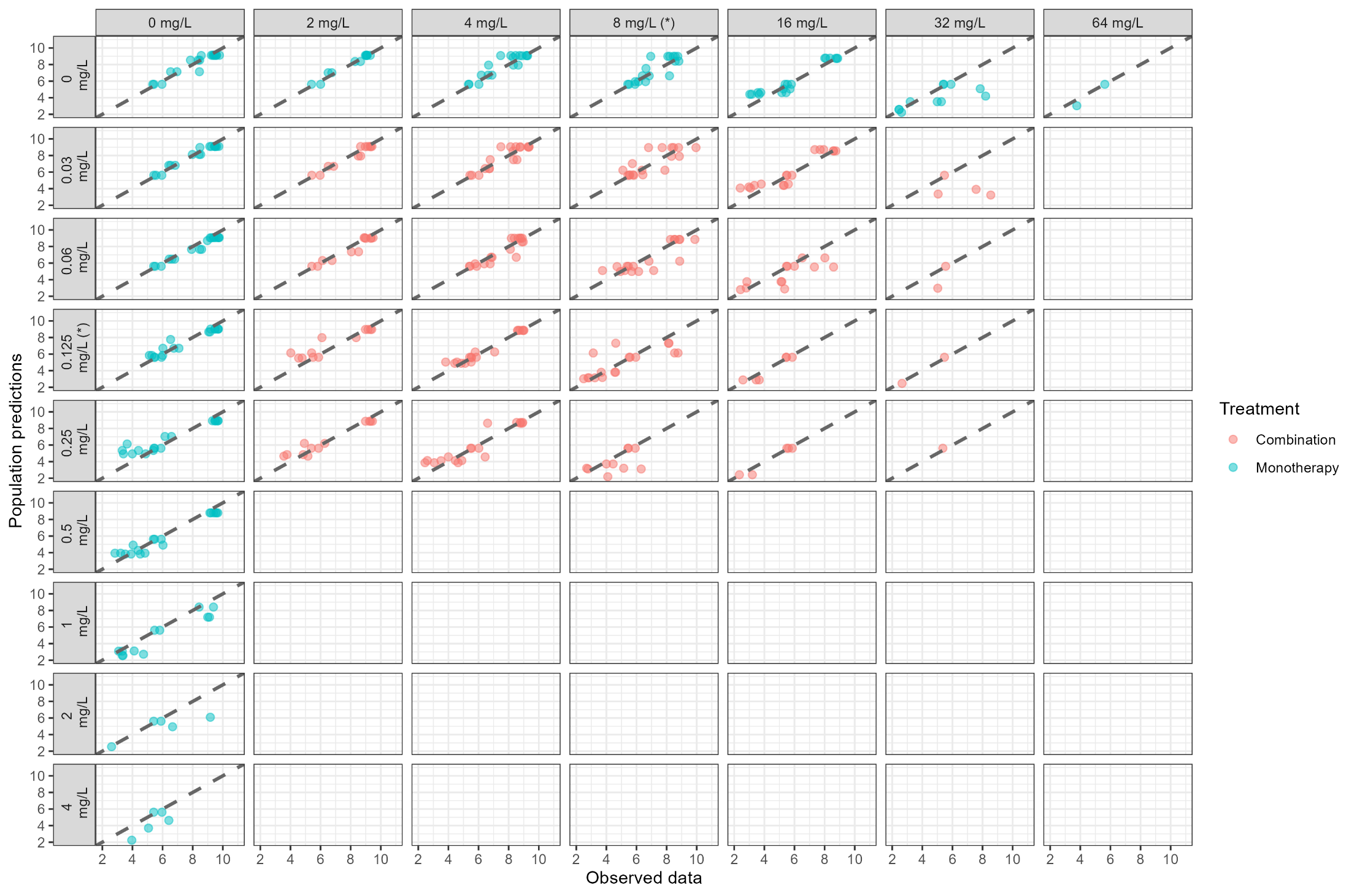

Figure S11. Observations vs predictions stratified by concentrations for *K. pneumoniae* NARA1584 isolate**

**Table S1. MIC and pharmacodynamic interaction between CZA and CST against *K. pneumoniae* strains**

| **Specie** | **Origin** | **Resistance** | **CZA MIC (mg/L)** | **CST MIC (mg/L)** | **Interaction type** | **Affected PD-parameter** | **Perpetrator drug** | **Estimated INT parameter**  **[95%CI]** |
| --- | --- | --- | --- | --- | --- | --- | --- | --- |
| *K. pneumoniae* | Isogenic | WT | 0.06 | 0.25 | Synergy | EC_50_ | CST | - 27% [-15%, -39%] |
| *K. pneumoniae* | Isogenic | OXA-48 | 0.06 | 0.25 | Asymmetry | EC_50_ | CZA CST | -4% [-2%, -6%] +126% [22%, 231%] |
| *K. pneumoniae* | Isogenic | KPC-2 | 0.06 | 0.125 | Additivity | None |  |  |
| *K. pneumoniae* | Isogenic | KPC-3 | 0.25 | 0.25 | Synergy | EC_50_ | CZA | -9% [-4%, -15%] |
| *K. pneumoniae* | SAK_MAP | OXA-48  CTX-M-14 | 1 | 0.25 | Synergy | EC_50_ | CZA | -39% [-16%, -62%] |
| *K.pneumoniae* | N1082 | Bla KPC-2 | 2 | 0.25 | Additivity | None |  |  |
| *K.pneumoniae* | R307 | Bla KPC-2 | 4 | 0.125 | Additivity | None |  |  |
| *K.pneumoniae* | 2977 | Bla KPC-2 | 2 | 32 | Synergy | EC_50_ | CST | -78% [-70%, -95%] |
| *K.pneumoniae* | NARA1174 | KPC-3 | 2 | 0.125 | Additivity | None |  |  |

MIC: minimum inhibitory concentration; CZA: ceftazidime/avibactam (fixed at 4 mg/L), EUCAST breakpoint > 8 mg/L; CST: colistin, EUCAST breakpoint > 2 mg/L. The four *K. pneumoniae* isolates in bold were studied in *in vitro* time kill experiments.

The pharmacodynamic interaction between CZA and CST was investigated against 9 carbapenemase producing isogenic or clinical *K. pneumoniae* strains, following the previous methodology [1, 2]. Dynamic checkerboard experiments with optimised drug concentrations were performed (n = 2) with CFU assessment at 24h. The pharmacodynamic interaction between CZA and CST was assessed using the GPDI model based on the estimated INT parameters [3]. INT = 0 (with 95%CI of the estimated INT parameters including 0) indicated additive interaction, INT < 0 indicated synergistic interaction (decrease of the EC50 of the victim drug) and INT > 0 indicated antagonistic interaction (increase of the EC50 of the victim drug). INT values of different polarities indicated an asymmetric interaction with concentration-dependent synergy and/or antagonism on the effect level.

1. Aubry R, Buyck J, Wicha SG, Decousser JW, Nordmann P, Couet W, Grégoire N. Systematic interaction screening of ceftazidime-avibactam and colistin against carbapenem-producing *Escherichia coli* and *Klebsiella pneumoniae*. 32^nd^ European Congress of Clinical Microbiology and Infectious Diseases (ECCMID), poster P0716.
2. Kroemer N, Aubry R, Grégoire N, Couet W, Wicha SG. Development and evaluation of D-optimal 2x2 checkerboard designs for identification of pharmacodynamic drug interactions. PAGE 29 (2021) Abstr 9696. 2021. <https://www.page-meeting.org/?abstract=9696>.
3. Wicha, S. G.; Chen, C.; Clewe, O.; Simonsson, U. S. H. A General Pharmacodynamic Interaction Model Identifies Perpetrators and Victims in Drug Interactions. *Nat Commun* 2017, *8*, 2129. <https://doi.org/10.1038/s41467-017-01929-y>.

**Code S1. NONMEM control stream of the expected additivity model for the NARA1295**

;; 2. Description: CZA + CST monotherapies

;; x1. Author: RAU

$PROBLEM In-vitro effect of the combination Ceftazidime/Avibactam + Colistin on the K. pneumoniae 334 N1174

$INPUT ID CAZ_CONC CST_CONC TIME DV CMT AMT EVID MDV BLQ INOC_th INOC_obs EXPID=DROP

$DATA I:/2020-COPROTECT/Data/Clean/Time_Kill_Curve/Combination_CZA-CST/TK_334_CZA-CST_dataset_mono.csv IGNORE=I

$SUBROUTINES ADVAN13 TOL=9

$MODEL

COMP=(CAZ_CONC) ; Ceftazidime concentration (µg/mL) - Avibactam at fixed concentration (4 µg/mL)

COMP=(CST_CONC) ; Colistin concentration (µg/mL)

COMP=(BTOT) ; Total bacteria

COMP=(ARON_CZA) ; Adapted fraction to CZA

COMP=(AROFF_CZA) ; Non adapated fraction to CZA

COMP=(ARON_CST) ; Adapted fraction to CST

COMP=(AROFF_CST) ; Non adapated fraction to CST

$PK

INOC=THETA(1) ; Estimated inoculum size (log10(CFU/mL))

KG=THETA(2) ; Bacterial growth rate constant (1/h)

BMAX=THETA(3) ; Bacterial count in the stationary phase (log10 (CFU/mL))

EMAX_CZA=THETA(4) ; Maximum killing rate of CZA effect (h-1)

EC50_CZA=THETA(5) ; CZA concentration needed to reach 50% of EMAX (mg/L)

GAMMA_CZA=THETA(6) ; Hill's factor

KON_CZA=THETA(7) ; Rate of adaptation development to CZA(1/h)

KOFF=THETA(8) ; Rate of adaptation reversal (l/h) fixed to 0

AR_CZA=THETA(9) ; ARmax_CZA = Fractional reduction of EMAX_CZA when bacteria are adapated

EMAX_CST=THETA(10) ; Maximum killing rate of CST effect (h-1)

EC50_CST=THETA(11) ; CST concentration needed to reach 50% of EMAX (mg/L)

GAMMA_CST=THETA(12) ; Hill's factor

KON_CST=THETA(13) ; Rate of adaptation development to CST(1/h)

AR_CST=THETA(14) ; ARmax_CST = Fractional reduction of EMAX_CST when bacteria are adapated

; Initial conditions

IF(A_0FLG.EQ.1) THEN

A_0(1)=CAZ_CONC

A_0(2)=CST_CONC

A_0(3)=10**INOC

A_0(4)=0

A_0(5)=1

A_0(6)=0

A_0(7)=1

ENDIF

$DES

; Logistic bacterial growth model

PLATEAU=1-(A(3)/10**BMAX)

; Adaptation to Ceftazidime/Avibactam

ADAPT_CZA=0

IF(A(1).GT.0) ADAPT_CZA=KON_CZA

E_ADAPT_CZA=AR_CZA*A(4) ; Residual effect of CZA against adapated bacteria

; Adaptation to Colistin

ADAPT_CST=0

IF(A(2).GT.0) ADAPT_CST=KON_CST

E_ADAPT_CST=AR_CST*A(6) ; Residual effect of CST against adapated bacteria

; Ceftazidime/Avibactam drug effect

KILL_CZA=0

IF(A(1).GT.0) KILL_CZA=(EMAX_CZA*(1-E_ADAPT_CZA)*A(1)**GAMMA_CZA)/(EC50_CZA**GAMMA_CZA+A(1)**GAMMA_CZA) ; Killing effect of CZA

; Colistin drug effect

KILL_CST=0

IF(A(2).GT.0) KILL_CST=(EMAX_CST*(1-E_ADAPT_CST)*A(2)**GAMMA_CST)/(EC50_CST**GAMMA_CST+A(2)**GAMMA_CST) ; Killing effect of CST

; Bliss Independence

IF(EMAX_CZA.GT.EMAX_CST) EMAX=EMAX_CZA

IF(EMAX_CZA.LT.EMAX_CST) EMAX=EMAX_CST

E_COMB=((KILL_CZA/EMAX)+(KILL_CST/EMAX)-((KILL_CZA/EMAX)*(KILL_CST/EMAX)))*EMAX ; Effect is normalised on EMAX to remain between 0 and 1 and then rescaled

; ODE

DADT(1)=0 ; Ceftazidime concentration constant over time

DADT(2)=0 ; Colistin concentration constant over time

DADT(3)=(KG*PLATEAU - E_COMB)*A(3) ; Total bacteria over time

DADT(4)=ADAPT_CZA*A(5)-KOFF*A(4) ; Adapted fraction over time

DADT(5)=KOFF*A(4)-ADAPT_CZA*A(5) ; Non adapted fraction over time

DADT(6)=ADAPT_CST*A(7)-KOFF*A(6) ; Adapted fraction over time

DADT(7)=KOFF*A(6)-ADAPT_CST*A(7) ; Non adapted fraction over time

$THETA

(5.64) ; INOC

(1.47) ; KG

(9.56) ; BMAX

(4.76) ; EMAX_CZA

(12.2) ; EC50_CZA

(2.01) ; GAMMA_CZA

(0.236) ; KON_CZA

(0) FIX ; KOFF

(0.719) ; AR_CZA

(13.2) ; EMAX_CST

(0.296) ; EC50_CST

(1.08) ; GAMMA_CST

(0.958) ; KON_CST

(0.918) ; AR_CST

$SIGMA 0.5 ; SIGMA

$OMEGA 0 FIX ; IIV_KON_CZA

$ERROR

A1=A(1)

A2=A(2)

A3=A(3)

A4=A(4)

A5=A(5)

A6=A(6)

A7=A(7)

BTOT_ERR = A3

IF(BTOT_ERR<1e-6) BTOT_ERR=1e-6

;Sim_start

IF(CMT.EQ.3.AND.BLQ.EQ.1) THEN ;M3 method for data BLQ

IPRED = LOG10(BTOT_ERR)

W= SQRT(SIGMA(1))

IRES = -9999

IWRES = -9999

F_FLAG=1

MDVRES=1

LOQ=LOG10(200) ;; LOQ = 200 CFU/mL

DUM=(LOQ-IPRED)/(W+0.00001)

CUMD=PHI(DUM) ;; PHI = cumulative density function

Y = CUMD

ENDIF

IF(CMT.EQ.3.AND.BLQ.EQ.0) THEN

;Sim_end

;Sim_start

;IF(CMT.EQ.3) THEN

;Sim_end

IPRED = LOG10(BTOT_ERR)

W= SQRT(SIGMA(1))

IRES = DV-IPRED

IWRES = (IRES/(W+0.00001))

;Sim_start

F_FLAG=0

MDVRES=0

;Sim_end

ERR1= EPS(1)

Y= IPRED+ERR1

ENDIF

$ESTIMATION METHOD=1 INTERACTION LAPLACIAN MAXEVAL=9999 SIGDIG=3 SIGL=9 SIGLO=9 PRINT=5 NOABORT POSTHOC

$COVARIANCE PRINT=E UNCONDITIONAL

$TABLE ID TIME CAZ_CONC CST_CONC DV CMT AMT BLQ A1 IPRED IWRES EVID CWRES ONEHEADER NOPRINT FILE=sdtab29

$TABLE ID TIME A1 A2 ADAPT_CZA A4 A5 E_ADAPT_CZA ADAPT_CST A6 A7 E_ADAPT_CST KILL_CZA KILL_CST A3 IPRED ONEHEADER NOPRINT FILE=patab29.csv FORMAT=,F12.2

**Code S2. NONMEM control stream of the observed interaction model for the NARA1295**

;; 2. Description: INT2_CST

;; x1. Author: RAU

$PROBLEM In-vitro effect of the combination Ceftazidime/Avibactam + Colistin on the K. pneumoniae 334

$INPUT ID CAZ_CONC CST_CONC TIME DV CMT AMT EVID MDV BLQ INOC_th INOC_obs EXPID=DROP

$DATA I:/2020-COPROTECT/Data/Clean/Time_Kill_Curve/Combination_CZA-CST/TK_334_CZA-CST_dataset1.csv IGNORE=I

$SUBROUTINES ADVAN13 TOL=9

$MODEL

COMP=(CAZ_CONC) ; Ceftazidime concentration (µg/mL) - Avibactam at fixed concentration (4 µg/mL)

COMP=(CST_CONC) ; Colistin concentration (µg/mL)

COMP=(BTOT) ; Total bacteria

COMP=(ARON_CZA) ; Adapted fraction to CZA

COMP=(AROFF_CZA) ; Non adapated fraction to CZA

COMP=(ARON_CST) ; Adapted fraction to CST

COMP=(AROFF_CST) ; Non adapated fraction to CST

$PK

INOC=THETA(1) ; Estimated inoculum size (log10(CFU/mL))

KG=THETA(2) ; Bacterial growth rate constant (1/h)

BMAX=THETA(3) ; Bacterial count in the stationary phase (log10 (CFU/mL))

EMAX_CZA=THETA(4) ; Maximum killing rate of CZA effect (h-1)

EC50_CZA=THETA(5) ; CZA concentration needed to reach 50% of EMAX (mg/L)

GAMMA_CZA=THETA(6) ; Hill's factor

KON_CZA=THETA(7) ; Rate of adaptation development to CZA(1/h)

KOFF=THETA(8) ; Rate of adaptation reversal (l/h) fixed to 0

AR_CZA=THETA(9) ; ARmax_CZA = Fractional reduction of EMAX_CZA when bacteria are adapated

EMAX_CST=THETA(10) ; Maximum killing rate of CST effect (h-1)

EC50_CST=THETA(11) ; CST concentration needed to reach 50% of EMAX (mg/L)

GAMMA_CST=THETA(12) ; Hill's factor

KON_CST=THETA(13) ; Rate of adaptation development to CST(1/h)

AR_CST=THETA(14) ; ARmax_CST = Fractional reduction of EMAX_CST when bacteria are adapated

INT=THETA(15) ; Interaction parameter = maximal fractional change of the affected PD parameter of the victim drug due to the interaction

INT_EC50=THETA(16) ; Concentration to reach half of INT (mg/L)

INT_GAMMA=THETA(17) ; Sigmoidicity parameter of the interaction relationship

; Initial conditions

IF(A_0FLG.EQ.1) THEN

A_0(1)=CAZ_CONC

A_0(2)=CST_CONC

A_0(3)=10**INOC

A_0(4)=0

A_0(5)=1

A_0(6)=0

A_0(7)=1

ENDIF

$DES

; Logistic bacterial growth model

PLATEAU=1-(A(3)/10**BMAX)

; Adaptation to Ceftazidime/Avibactam

ADAPT_CZA=0

IF(A(1).GT.0) ADAPT_CZA=KON_CZA

E_ADAPT_CZA=AR_CZA*A(4) ; Residual effect of CZA against adapated bacteria

; Adaptation to Colistin

ADAPT_CST=0

IF(A(2).GT.0) ADAPT_CST=KON_CST

E_ADAPT_CST=AR_CST*A(6) ; Residual effect of CST against adapated bacteria

; GPDI model (to be adapted accordingly to the interaction)

INTERACTION=0 ; no interaction in single drug experiment

IF(A(1).GT.0.AND.A(2).GT.0) INTERACTION=(INT*A(2)**INT_GAMMA)/(INT_EC50**INT_GAMMA+A(2)**INT_GAMMA) ; GPDI model describing PD interaction

; Ceftazidime/Avibactam drug effect

KILL_CZA=0

IF(A(1).GT.0) KILL_CZA=(EMAX_CZA*(1-E_ADAPT_CZA)*A(1)**GAMMA_CZA)/((EC50_CZA*(1+INTERACTION))**GAMMA_CZA+A(1)**GAMMA_CZA) ; Killing effect of CZA, modified by INTERACTION

; Colistin drug effect

KILL_CST=0

IF(A(2).GT.0) KILL_CST=(EMAX_CST*(1-E_ADAPT_CST)*A(2)**GAMMA_CST)/(EC50_CST**GAMMA_CST+A(2)**GAMMA_CST) ; Killing effect of CST

; Bliss Independence

IF(EMAX_CZA.GT.EMAX_CST) EMAX=EMAX_CZA

IF(EMAX_CZA.LT.EMAX_CST) EMAX=EMAX_CST

E_COMB=((KILL_CZA/EMAX)+(KILL_CST/EMAX)-((KILL_CZA/EMAX)*(KILL_CST/EMAX)))*EMAX ; Effect is normalised on EMAX to remain between 0 and 1 and then rescaled

; ODE

DADT(1)=0 ; Ceftazidime concentration constant over time

DADT(2)=0 ; Colistin concentration constant over time

DADT(3)=(KG*PLATEAU - E_COMB)*A(3) ; Total bacteria over time

DADT(4)=ADAPT_CZA*A(5)-KOFF*A(4) ; Adapted fraction over time

DADT(5)=KOFF*A(4)-ADAPT_CZA*A(5) ; Non adapted fraction over time

DADT(6)=ADAPT_CST*A(7)-KOFF*A(6) ; Adapted fraction over time

DADT(7)=KOFF*A(6)-ADAPT_CST*A(7) ; Non adapted fraction over time

$THETA

(5.54) ; INOC

(1.89) ; KG

(9.18) ; BMAX

(4.93) ; EMAX_CZA

(10.7) ; EC50_CZA

(1.84) ; GAMMA_CZA

(0.214) ; KON_CZA

(0) FIX ; KOFF

(0.642) ; AR_CZA

(13.1) ; EMAX_CST

(0.256) ; EC50_CST

(0.97) ; GAMMA_CST

(0.948) ; KON_CST

(0.873) ; AR_CST

(-0.1) ; INT

(0.256) ; INT_EC50

(10) FIX ; INT_GAMMA

$SIGMA 0.5 ; SIGMA

$OMEGA 0 FIX ; IIV_KON_CZA

$ERROR

A1=A(1)

A2=A(2)

A3=A(3)

A4=A(4)

A5=A(5)

A6=A(6)

A7=A(7)

BTOT_ERR = A3

IF(BTOT_ERR<1e-6) BTOT_ERR=1e-6

;Sim_start

IF(CMT.EQ.3.AND.BLQ.EQ.1) THEN ;M3 method for data BLQ

IPRED = LOG10(BTOT_ERR)

W= SQRT(SIGMA(1))

IRES = -9999

IWRES = -9999

F_FLAG=1

MDVRES=1

LOQ=LOG10(200) ;; LOQ = 200 CFU/mL

DUM=(LOQ-IPRED)/(W+0.00001)

CUMD=PHI(DUM) ;; PHI = cumulative density function

Y = CUMD

ENDIF

IF(CMT.EQ.3.AND.BLQ.EQ.0) THEN

;Sim_end

;Sim_start

;IF(CMT.EQ.3) THEN

;Sim_end

IPRED = LOG10(BTOT_ERR)

W= SQRT(SIGMA(1))

IRES = DV-IPRED

IWRES = (IRES/(W+0.00001))

;Sim_start

F_FLAG=0

MDVRES=0

;Sim_end

ERR1= EPS(1)

Y= IPRED+ERR1

ENDIF

$ESTIMATION METHOD=1 INTERACTION LAPLACIAN MAXEVAL=9999 SIGDIG=3 SIGL=9 SIGLO=9 PRINT=5 NOABORT POSTHOC

$COVARIANCE PRINT=E UNCONDITIONAL

$TABLE ID TIME CAZ_CONC CST_CONC DV CMT AMT BLQ A1 IPRED IWRES EVID CWRES ONEHEADER NOPRINT FILE=sdtab124

$TABLE ID TIME A1 A2 ADAPT_CZA A4 A5 E_ADAPT_CZA ADAPT_CST A6 A7 E_ADAPT_CST INTERACTION KILL_CZA KILL_CST A3 IPRED ONEHEADER NOPRINT FILE=patab124.csv FORMAT=,F12.2
